# Comprehensive characterization of toxins during progression of inhalation anthrax in a non-human primate model

**DOI:** 10.1371/journal.ppat.1010735

**Published:** 2022-12-19

**Authors:** Anne E. Boyer, Maribel Gallegos-Candela, Renato C. Lins, Maria I. Solano, Adrian R. Woolfitt, John S. Lee, Daniel C. Sanford, Katherine A. B. Knostman, Conrad P. Quinn, Alex R. Hoffmaster, James L. Pirkle, John R. Barr

**Affiliations:** 1 Centers for Disease Control and Prevention, Atlanta, Georgia, United States of America; 2 Battelle Atlanta Analytical Services, Atlanta, Georgia, United States of America; 3 Biomedical Advanced Research and Development Authority, Washington, DC, United States of America; 4 Battelle Biomedical Research Center, West Jefferson, Ohio, United States of America; Northwestern University Feinberg School of Medicine, UNITED STATES

## Abstract

Inhalation anthrax has three clinical stages: early-prodromal, intermediate-progressive, and late-fulminant. We report the comprehensive characterization of anthrax toxins, including total protective antigen (PA), total lethal factor (LF), total edema factor (EF), and their toxin complexes, lethal toxin and edema toxin in plasma, during the course of inhalation anthrax in 23 cynomolgus macaques. The toxin kinetics were predominantly triphasic with an early rise (phase-1), a plateau/decline (phase-2), and a final rapid rise (phase-3). Eleven animals had shorter survival times, mean±standard deviation of 58.7±7.6 hours (fast progression), 11 animals had longer survival times, 113±34.4 hours (slow progression), and one animal survived. Median (lower–upper quartile) LF levels at the end-of-phase-1 were significantly higher in animals with fast progression [138 (54.9–326) ng/mL], than in those with slow progression [23.8 (15.6–26.3) ng/mL] (p = 0.0002), and the survivor (11.1 ng/mL). The differences were also observed for other toxins and bacteremia. Animals with slow progression had an extended phase-2 plateau, with low variability of LF levels across all time points and animals. Characterization of phase-2 toxin levels defined upper thresholds; critical levels for exiting phase-2 and entering the critical phase-3, 342 ng/mL (PA), 35.8 ng/mL (LF), and 1.10 ng/mL (EF). The thresholds were exceeded earlier in animals with fast progression (38.5±7.4 hours) and later in animals with slow progression (78.7±15.2 hours). Once the threshold was passed, toxin levels rose rapidly in both groups to the terminal stage. The time from threshold to terminal was rapid and similar; 20.8±7.4 hours for fast and 19.9±7.5 hours for slow progression. The three toxemic phases were aligned with the three clinical stages of anthrax for fast and slow progression which showed that anthrax progression is toxin- rather than time-dependent. This first comprehensive evaluation of anthrax toxins provides new insights into disease progression.

## Author summary

This first analysis of all major anthrax toxins during inhalation anthrax revealed three stages of toxemia that align with the three clinical stages, early-, intermediate-, and late-stage anthrax. High toxin levels in the early stages led to faster progression and lower levels to slower progression. Critical toxin levels were defined for entering the late-stage where treatment usually fails. This study provides new details of anthrax progression which has the potential to improve treatment and save lives.

## Introduction

*Bacillus anthracis* is a gram positive, spore-forming bacterium and the causative agent of anthrax [1]. Anthrax can take many forms, cutaneous, gastrointestinal, injection, and the most lethal, inhalation. As such, *B*. *anthracis* is a U.S. Category A priority pathogen, and Tier 1 select agent (www.selectagents.gov), with the potential for aerosol dispersion and mass casualties [2]. Historically, the fatality rate of untreated inhalation anthrax ranged from 89%-100% [3–6]. In 2001, anthrax spores were intentionally distributed through the U.S. mail. Even with the use of antimicrobial agents and modern intensive medical support, the fatality rate was 45% [7–9]. The high rate of fatality is due to the production of two binary toxins that disable host’s immune response, cause endothelial barrier dysfunction, edema, and hemorrhage [10]. Even after the clearance of bacilli by antimicrobial treatment, the toxins persists for days, causing further damage and death [11]. A better understanding of the levels of anthrax toxins during the stages of infection would provide insights into their role in pathogenicity.

The two major *B*. *anthracis* toxins consist of binary combinations of protective antigen (PA), which binds enzymatic factors, lethal factor (LF) and edema factor (EF), forming active toxin complexes lethal toxin (LTx) and edema toxin (ETx), respectively [12]. PA is secreted as an 83-kDa protein (PA83) that binds cell surface receptors TEM8 and CMG2 [13,14]. There it is hydrolyzed by furin-like proteases to 63 kDa (PA63), which causes it to form heptamers/octamers that readily bind LF and EF, forming the LTx and ETx complexes [15–18]. PA83 is also hydrolyzed by serum proteases, forming PA63, and ultimately, LTx and ETx [19–23]. LF, a 90-kDa zinc-dependent endoproteinase, hydrolyzes and inactivates several members of the mitogen-activated protein kinase kinase (MAPKK) family of proteins that are central to various cell signaling responses [24]. EF, an 89-kDa calcium and calmodulin dependent adenylate cyclase, has a high rate of catalysis of ATP to cyclic AMP [25]. The accumulation of cyclic AMP contributes locally to edema, but also activates protein kinase A and exchange protein, both of which control a variety of cellular functions [10]. In early stages of infection, LTx and ETx synergistically suppress innate immune responses, allowing the infection to take hold, bacteria to proliferate, and toxins to accumulate [10,26–28]. In later stages the toxins contribute to disruption of endothelial barrier integrity, causing edema, pleural effusions, hemorrhage, and hemodynamic instability [29–31]. After a certain point, the damage becomes insurmountable and largely resistant to standard procedures of cardiovascular support [32]. It is not yet known what toxin levels correlate with these stages and their clinical impact.

Clinically, inhalation anthrax has been described as having three-stages: 1) early-prodromal, 2) intermediate-progressive, and 3) late-fulminant [33,34]. The first stage presents with non-specific symptoms, often resembling the flu. The cure rate is highest in this early stage, but since anthrax is rare, a low index of suspicion at clinical presentation can delay appropriate treatment. In the second stage, non-specific symptoms continue but pathology and the clinical signs progress. The potential for survival is still high in this stage but requires aggressive medical intervention. Onset of the third stage is rapid with respiratory failure, shock, and often meningitis. The likelihood of survival is low in the third stage. This was most evident during the anthrax letter attacks of 2001. All 11 cases with inhalation anthrax received aggressive medical intervention. However, six that presented during stage-2 survived, while five presenting in stage-3 did not [7,8,34]. Novel treatments such as antitoxins developed in more recent years may help to improve outcomes [35]. However, more needs to be learned about the progression of anthrax to help discern stages, which might aid in understanding responses to treatment and when it fails.

Of the animal models for inhalation anthrax, non-human primates (NHP), both rhesus macaques and cynomolgus macaques have been extensively studied for anthrax pathology, therapeutics, and vaccines [36–43]. However, of the many toxins and biomarkers of anthrax, only PA and bacteremia have been characterized in cynomolgus macaques [42]. We have developed and validated sensitive and specific mass spectrometry (MS) methods for all major anthrax toxin proteins and their toxin complexes. They include methods for total-LF (LF+LTx) [44], LTx (PA-LF) [22], total-EF (EF+ETx), ETx (PA-EF) [23], and PA (total PA, full length PA83, and PA63) [45]. Application of the methods in five rhesus macaques showed a triphasic kinetics of toxemia [22,23,45,46]. These were too few and limited in scope to draw major conclusions about staging. Here, these MS methods were applied to measure and characterize anthrax toxins over the course of infection in the cynomolgus macaque NHP model. This study provides the first comprehensive assessment of all the major anthrax toxins during infection which, along with traditionally measured bacteremia, addresses the complexities of anthrax progression, yielding insights that may improve medical management and public health outcomes during an exposure event.

## Results

Detailed data with associated information is available for all tables and figures where it is not already included in the main article (S1 Data File).

### 1. Cynomolgus macaque study outcomes

*Exposures and clinical outcomes*. The study included 23 cynomolgus macaques, 12 males and 11 females, with a mean±standard deviation weight of 4.0±0.61 kg at challenge (Table 1). The weight was 3.7±0.48 kg for females and 4.3±0.61 kg for males. The NHP’s were challenged in two groups. Group-1 included 10 animals with two non-challenged controls, for 8 challenged in group-1. The two controls did not develop disease or detectable toxemia/bacteremia. Group-2 included the two control animals from group-1 which were challenged with 13 others, for 15 animals. The collective exposure dose of 221(±29.2) Ames LD_50_ and that of both group-1 (218±32 LD_50_) and group-2 (223±29 LD_50_) were similar. There were 22 non-survivors, ordered by survival time which ranged from 48.5–201.9 hours-h (Table 1 and S1 Fig, survival curve). One animal survived to the end-of-study. When adjusted by weight, the Ames LD_50_ exposure dose (LD_50_/kg) tended to be higher in animals that had shorter survival times with some exceptions. The dose for animal 1 with the shortest survival time (48 LD_50_/kg) was similar to that for animal 23 that survived (49 LD_50_/kg), and lower than that for the animal 22 with the longest survival time (60 LD_50_/kg). The mean±standard deviation spore dose/kg of the 11 animals with shorter survival times was 61.6±11.7 LD_50_/kg and for the 11 non-survivors with longer survival was 51.9±11.0 LD_50_/kg. These differences did not meet the alpha level of 0.05 for significance (p = 0.0583). A longer time to death does not clearly correlate with lower doses.

**Table 1:** Cynomolgus macaques, challenge, symptoms, and survival time.

	Animal ID	Gender	Weight (Kg) Day 0	Ames LD_50_	LD_50_ per kg	Symptom Onset-h	First Symptom	Hours to Death (Days)	Manner of Death	Pathology Cause of Death
1	C58282^1^	M	4.6	219	**48**	29.1	HP	48.5 (2.0)	Euth	Sepsis
2	C59621	F	3.2	230	72	47.4	N/Eu	48.9 (2.0)	FD	Sepsis
3	C60763	M	3.3	200	61	54.7	FD	54.7 (2.3)	FD	Sepsis
4	C58176	M	4.2	280	67	38.2	W	55.2 (2.3)	FD	Sepsis
5	C58003^**+**^	F	4.2	232	55	40.3	HP	57.3 (2.4)	FD	Sepsis
6	C59656	F	3.7	206	56	40.9	HP	57.9 (2.4)	FD	Sepsis
7	C58214^1^	F	3.4	246	72	28.5	HP	58.4 (2.4)	FD	Sepsis
8	C60732	M	3.1	258	83	35.8	HP	59.0 (2.5)	Euth	Sepsis
9	C59669	F	3.4	223	66	56.3	HP, L	64.8 (2.7)	Euth	Sepsis
10	C57161^1^	F	3.7	203	55	41.5	HP	66.9 (2.8)	FD	Sepsis
11	C58170^**+**^	M	4.8	208	43	61.4	HP	74.1 (3.1)	Euth	Sepsis
12	C59619	F	3.6	270	75	35.3	W, HP, V	76.4 (3.2)	FD	Sepsis
13	C58135	M	4.0	186	47	34.9	HP	90.4 (3.8)	Euth	Sepsis
14	C57793	M	4.1	187	46	67.0	FA	91.1 (3.8)	FD	Sepsis
15	C58167	M	4.9	215	44	37.3	HP, W	91.7 (3.8)	FD	Sepsis
16	C57998^1^	F	3.5	199	57	77.2	L, W	100.8 (4.2)	FD	Sepsis
17	C59617	F	4.9	203	41	88.1	HP	105.1 (4.4)	FD	Meningitis/ Sepsis
18	C58159^1^	M	4.2	188	45	30.2	HP	109.0 (4.5)	Euth	Meningitis
19	C58122^1^	M	4.8	181	38	35.6	DI	115.1 (4.8)	FD	Sepsis
20	C57997^1^	F	3.9	234	60	53.9	W	125.9 (5.2)	FD	Sepsis
21	C58156^1^	M	4.7	274	58	60.7	HP	140.1 (5.8)	FD	Meningitis
22	C59618	F	3.4	202	60	57.0	HP	201.9 (8.4)	Euth	Sepsis
23	C58174	M	4.9	239	**49**	108.4	FA	336.6 (14)	EOS	NA
	Mean±SD		4.0±0.61	221±29.2	56.4±12.0	50.4±20.2		86.1±37.1		

23 animals were challenged in two groups. The first group included 8 challenged animals^1^ and 2 non-challenged controls. The second group included 15 animals, with the 2 controls from the first group^**+**^. SD-standard deviation. LD_50_/Kg in bold font show similar exposure doses for animal 1 and 23. *Survivor sacrificed at the end-of-study (EOS), 14 days. Animals were observed every 6 hours for symptoms and criteria for euthanasia. Symptom onset is the time from challenge to first symptom; hunched posture (HP), wheezing (W), lethargic (L), vomiting (V), facial edema (FA) and diarrhea (DI), or none (N) until euthanized (E), found dead (FD). Euthanized (Euth), found dead (FD).

*Symptom onset*. The onset of symptoms has been represented by an increase in body temperature from normal or baseline (47). Body temperatures were monitored by telemetry before and after challenge. The increase in body temperature was not a consistent indicator of symptoms (S2 Fig). It was present in only 12 animals for the shoulder and 11 animals for the hip. When present, the elevated temperatures were transient, except in the shoulder of three animals with the longest survival time.

Symptoms were also monitored by veterinarians, every 6 hours after challenge. The first clinical sign(s) and the time of occurrence (symptom onset) was recorded (Table 1). The time from challenge to symptom onset ranged from 28.5 hours at the earliest, to 108.4 hours at the latest, in the survivor. Mean±standard deviation time to symptom onset was 50.4±20.2 hours. The most common first clinical sign was hunched posture, present in 15 animals and the least common was diarrhea and vomiting, present in only one animal each. Four animals had two or more symptoms at onset, while two had no clinical signs recorded before early death/euthanasia at 48.9- and 54.7-hours, suggesting the progression from normal behavior to death or moribund occurred in less than 6 hours.

*Cause of death*. Terminal microscopic and gross pathology, to be described later, provided the final determination of the cause of death as being due to either sepsis, for which bacteremia was found in multiple organs, or meningitis, with whole brain pathology and bacteremia limited to lymphatic organs (Table 1). Three animals with longer survival times had whole brain pathology; two of these were considered meningitis alone.

### 2. Anthrax toxemia and bacteremia

#### 2.1 Pre-symptom onset detection of anthrax biomarkers

**Fig 1:**
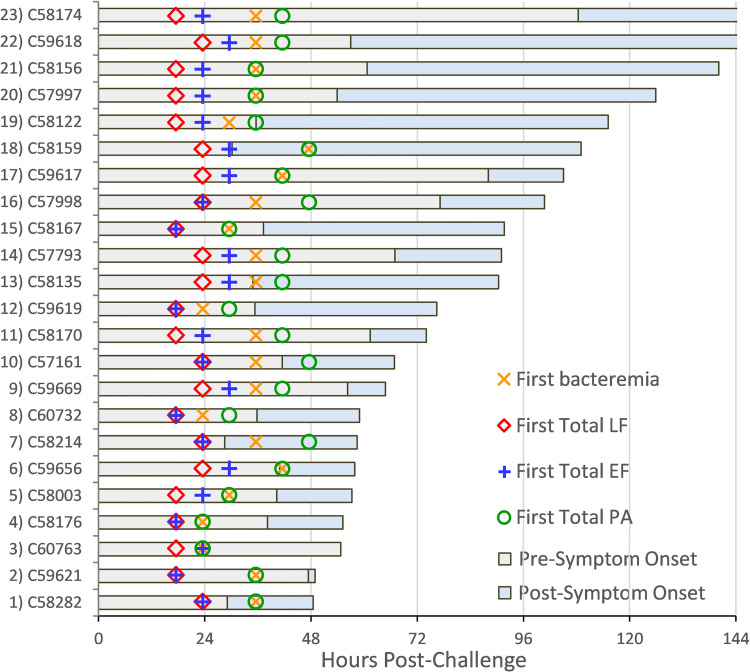
First detection of anthrax biomarkers. First detection of biomarkers relative to symptom onset in 23 cynomolgus macaques with inhalation anthrax. Pre-symptom onset period (gray bar) and post-symptom onset period (blue bar) out to 144 hours, for total lethal factor (LF), total edema factor (EF), total protective antigen (PA), and confirmed bacteremia. Detection limits for LF (0.0027 ng/mL), EF (0.00002 ng/mL), PA (1.87 ng/mL) and confirmed bacteremia (≥100 cfu/mL). Non-confirmed results of ‘+’ excluded here but included in Table 2.

The limits of detection for all the anthrax toxin methods are summarized in Table 2. Total-LF and -EF methods detect and measure the enzymatic activity which gives lower detection limits, whereas the PA method detects and measures the specific trypsin-digest peptide fragments of PA itself which has a higher detection limit. The earliest detection of total toxins LF, EF, and PA, and bacteremia were graphed relative to symptom onset. This showed that LF was the first biomarker detected, preceding symptom onset in all 23 animals (Fig 1). EF was the next biomarker detected, 6 hours after LF in 14, and before symptoms in 21 animals. Bacteremia and PA were detected later; before symptom onset in 19 and 16 animals, respectively.

**Table 2:** Anthrax toxin mass spectrometry (MS) 3-step methods summary.

Analyte	Pre-MS Sample Prep	LOD (ng/mL)
LF Total	IP-catalysis	0.0027
LTx	IP-catalysis	0.0075
EF Total	IP-catalysis	0.00002
ETx	IP-catalysis	0.00002
PA Total	IP-tryp digest	1.87
PA83	IP-tryp digest	1.22

Immunoprecipitation (IP), limit of detection (LOD), tryptic (tryp).

Table 3 includes the summary for pre-symptom onset detection for total toxins, toxin complexes, LTx and ETx, and full length PA83 for 1) the mean time to first detection, 2) the time detection preceded symptom onset, and 3) the percent of animals positive at early time points. LF was detected earliest, well before symptoms, in 100% of animals by 24 hours. EF was detected second earliest, in 65% by 24 hours and 100% by 30 hours. Detection of bacteremia followed. Toxin complexes, LTx and ETx, were detected about 9 hours after their total LF and total EF counterparts. Total PA was detected later, even though it was present earlier in the form of LTx and ETx. All toxins except for PA83 reached 100% positivity by 48 hours. Full length PA83 was the last to test positive and never reached 100% positivity at any time point.

**Table 3:** Pre-symptom onset detection.

Biomarker	Time first detected (h)	Time detected before symptoms (h)	Percent positive at post-challenge time				
			18h	24h	30h	36h	48h
**Total LF**	20.9±3.1	29.6±20.5	52.2	100.0	100	100	100
**Total EF**	24.5±4.4	25.9±19.8	21.7	65.2	100	100	100
**Bact NC (+)**	30.5±6.5	19.9±19.8	17.4	26.1	52.2	100	100
**LTx**	30.5±4.4	19.9±19.5	0	22.7	100	100	100
**ETx**	33.4±7.6	17.0±22.6	0	21.7	52.2	73.9	100
**Bact Confirmed**	34.2±6.1	16.2±19.7	0	17.4	30.4	87.0	100
**Total PA**	38.1±7.4	12.3±19.7	0	8.70	26.1	50.0	100
**PA83 (n = 21)**	59.9±39.1	-11.2±36.2	0	0	4.3	18.2	40.9

First appearance of anthrax toxins and bacteremia. Mean time (hours-h) each biomarker was first detected post-challenge and the mean time (h) the first detected occurred before symptom onset in each animal. The percent positive for each biomarker of 23 animals at all early time points (prior to the mean symptom onset at 50.4 hours). Bacteremia (Bact) was quantified by serial dilution, plating in triplicate and colony counting which has an LOD of 100 cfu/mL. Only bacteremia reported ≥100 cfu/mL were confirmed. Results below this were reported as + for two of three plates with less than 10 colonies. Inclusion of the non-confirmed (NC) + results were included for Bact NC (+).

#### 2.2 Biomarker levels at symptom onset

Symptom onset toxin levels and bacteremia were determined for each animal (Table 4). LF, LTx, and EF were positive at symptom onset in all 23 animals. Total PA was not detected in four animals with LTx (PA-LF) levels below 0.26 ng/mL. Bacteremia was detectable in 21 animals, ETx in 20, and total PA in 19 of the 23 animals. PA83 was only positive in 7 animals. Median symptom onset levels for all 23 animals were in the mid-low ng/mL for PA, LF, and LTx and sub-ng/mL for EF and ETx. The median for bacteremia was 1.3E+04 cfu/mL. The interquartile ranges demonstrate a broad range of concentrations. These are likely a result of the different rates of progression and stages during which symptoms were first observed. As related to progression rates, median symptom onset biomarker levels were higher in 11 animals with shorter survival. Specifically, median PA, LF, LTx, and bacteremia levels were 2.3-, 10-, 20-, and 22-fold higher, and EF and ETx were 74- and 62-fold higher, respectively, for the 11 animals with shorter survival than the 11 animals with longer survival. As related to time of symptom onset, the four animals with earliest symptoms (28.5–34.9 h) were those with the lowest LF, LTx and EF levels, and low or non-detectable PA, ETx, and bacteremia. In these four animals with earliest symptoms, LF ranged from 0.076–1.3 ng/mL. The next sample, 6 hours later, showed that LF increased to 0.538–6.4 ng/mL. PA and ETx remained non-detectable, 6-hours later, in the two animals with the lowest LF levels.

**Table 4:** Symptom onset biomarker levels.

Animal ID	Hours to term	Symptom Onset-h	Sample time-h	Bact (next/term*) (cfu/mL)	Total-LF (next) (ng/mL)	LTx (ng/mL)	Total-EF (ng/mL)	ETx (next) (ng/mL)	Total-PA (next) (ng/mL)	PA83 (ng/mL)
1) C58282	48.5	29.1	30	pos (5.3E+02)	0.235 (2.99)	0.103	0.00068	<LOD (0.0045)	<LOD (9.11)	<LOD
2) C59621	48.9	47.4	42˜	2.9E+06 (1.3E+09*)	57.8 (NA)	23.7	20.7	1.87	333 (NA)	67.4
3) C60763	54.7	54.7	48˜	5.4E+06 (6.2E+08*)	366 (NA)	203	74.7	15.7	2222 (NA)	259.4
4) C58176	55.2	38.2	42	9.1E+04 (3.1E+06)	133 (NA)	50.9	2.64	0.602	199 (NA)	7.7
5) C58003	57.3	40.3	42	5.9E+06 (2.6E+05)	300 (NA)	169	23.7	5.64	1143 (NA)	163
6) C59656	57.9	40.9	42	7.0E+03 (3.6E+06)	8.5 (75.2)	0.893	0.059	0.00082	13.6 (731)	<LOD
7) C58214	58.4	28.5	30	neg (3.0E+02)	0.136 (0.839)	0.058	0.0005	<LOD (<LOD)	<LOD (<LOD)	<LOD
8) C60732	59.0	35.8	36	7.4E+06 (2.9E+03)	313 (557)	165	39.2	7.1	1189 (693)	176
9) C59669	64.8	56.3	60	1.9E+05 (4.0E+08*)	200 (NA)	149	5.62	0.872	282 (NA)	<LOD
10) C57161	66.9	41.5	48	7.2E+06 (1.1E+06)	366 (741)	245	53.4	15	2221 (2303)	516
11) C58170	74.1	61.4	60˜	6.3E+04 (8.3E+08*)	189 (NA)	139	0.194	0.034	428 (NA)	<LOD
Mean-h Median Short	58.7±7.6	43.1±10.8	41.3±9.9	1.9E+05 (3.5E+04–5.7E+06)	189 (33.2–307)	139 (12.3–167)	5.6 (0.13–31.5)	0.87 (0.017–6.4)	333 (106–1166)	ND
12) C59619	76.4	35.3	36	1.8E+05 (1.5E+04)	32.5 (37.4)	12.5	0.661	0.278	368 (367)	36.2
13) C58135	90.4	34.9	36	2.5E+02 (5.3E+03)	1.30 (6.4)	0.256	0.0053	0.00035	<LOD (7.21)	<LOD
14) C57793	91.1	67.0	72	9.2E+04 (1.2E+08)	33.3 (7996)	15.8	0.933	0.272	275 (34,638)	<LOD
15) C58167	91.7	37.3	42	8.5E+03 (7.1E+03)	18.9 (19.4)	6.49	0.38	0.082	129 (65.0)	<LOD
16) C57998	100.8	77.2	84	2.9E+04 (3.1E+07)	22.6 (1754)	9.59	0.459	0.035	144 (15,503)	5.57
17) C59617	105.1	88.1	84	9.6E+03 (5.9E+04)	93.8 (178)	64.1	1.95	0.511	599 (813)	25.4
18) C58159	109.0	30.2	30	neg (pos)	0.076 (0.538)	0.029	0.00056	<LOD (<LOD)	<LOD (<LOD)	<LOD
19) C58122	115.1	35.6	36	8.4E+03 (3.6E+03)	5.33 (22.0)	1.29	0.044	0.00042	18.7 (152)	<LOD
20) C57997	125.9	53.9	60	3.7E+02 (5.3E+02)	15.5 (10.4)	6.97	0.048	0.014	200 (96.7)	<LOD
21) C58156	140.1	60.7	60	1.3E+04 (2.6E+02)	19.8 (11.7)	8.68	0.076	0.014	172 (137)	<LOD
22) C59618	201.9	57.0	60	9.2E+02 (pos)	8.51 (6.3)	1.05	0.021	0.00065	25.7 (28.3)	<LOD
Mean-h Median Longer	113±34.4	52.5±19.5	54.5±19.8	8.5E+03 (6.5E+02–2.1E+04)	18.9 (6.9–27.6)	7.0 (1.2–11.0)	0.076 (0.033–0.56)	0.014 (0.00054–0.18)	144 (22.2–238)	ND
23) C58174	EOS	108.4	108	3.4E+02 (neg)	7.3 (4.2)	1.6	0.041	0.0058	43.8	<LOD
Mean-h 22-NS Median (ng/mL) All 23	86.1±37.1	47.8±16.1	48.9±17.4	1.3E+04 (6.5E+02–1.9E+05)	22.6 (7.9–161)	9.6 (1.2–102)	0.38 (0.043–4.1)	0.035 (0.00074–0.74)	199 (22.2–398)	

Biomarker levels at the sample time (hours-h) closest to symptom onset for toxins (ng/mL) and bacteremia (cfu/mL), in cynomolgus macaques (CM) with inhalation anthrax. *˜* Last available sample was used when symptom onset sample was not available. Animals numbered by survival time (h). Symptom onset is the time from challenge to first observed symptom. Mean±standard deviation for hours (survivor excluded) and median and interquartile range (IQR) as median (lower–upper IQR) for all biomarker levels, except PA83, calculated in Microsoft Excel. For calculations, values <LOD were included at ½ the LOD per method (Table 2) and for bacteremia (Bact) a ‘+’ for low positive (pos) at 6 cfu/mL and ‘0’ negative (neg) at 3 cfu/mL [35]. Animals with earliest symptom onset (underlined). Measurements for the ‘next’ time point were included for ETx, bacteremia, LF, and PA levels 6–12 hours later; *bacteremia when the next measurement was at terminal time. Biomarkers were analyzed collectively (all, n = 23) and separately for animals with shorter survival (n = 11) and longer survival (n = 11). End of study (EOS). Not determined (ND).

#### 2.3 Kinetics of toxemia and bacteremia

**Fig 2:**
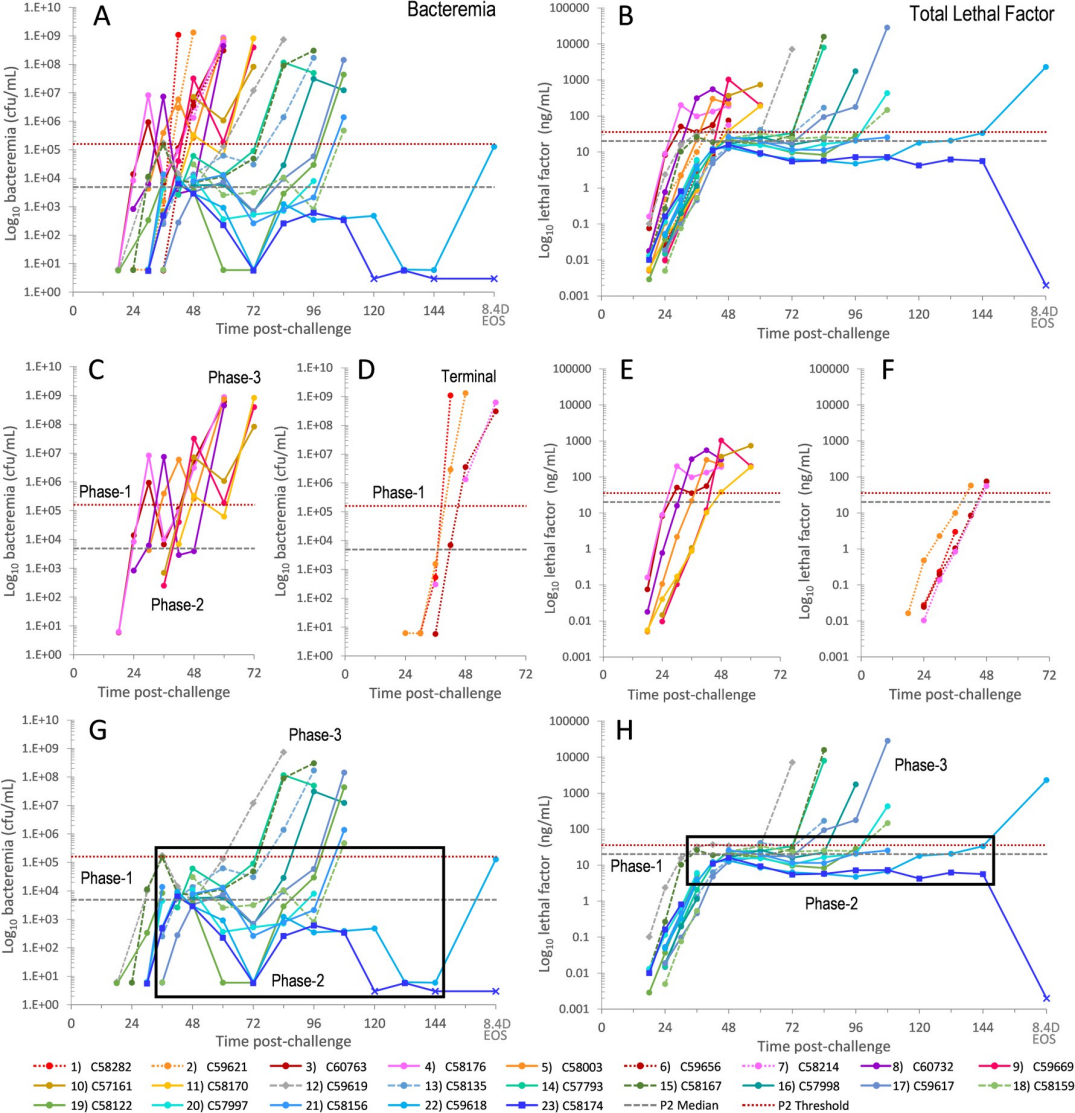
Kinetics of bacteremia and lethal factor in 23 cynomolgus macaques. Bacteremia (Bact) in (cfu/mL) (A) and total lethal factor (LF) in (ng/mL) (B) over the course of infection. 11 animals had higher early bacteremia/toxemia and shorter survival (fast progression) (red/orange/purple lines), and 11 animals had lower early toxemia and longer survival (slow progression) (aqua/green lines), and one survivor with low early toxemia (blue line, square symbols). Triphasic kinetics: initial rise (phase-1), plateau/decline (phase-2), and final rise to terminal (phase-3). Bacteremia and LF in 7 animals with fast triphasic kinetics (C) and (E) and 4 animals with monophasic kinetics, a single rapid rise, (D) and (F), respectively. Bacteremia and LF in 11 animals with slow progression and survivor, (G) and (H), respectively. Final time points for 22) C59618 that died at 8.4 days (8.4D) and the survivor, 23) C58174, at the end-of-study (EOS, 14-days) are not to scale. For bacteremia (bact), non-quantifiable results of low positive were assigned a value of 6 and negative at 3 cfu/mL. Bacteremia and LF below the limit of detection (x-symbol). Boxes represent the broad range of bacteremia in phase-2 (G) and narrow range of LF levels during phase-2 (H).

*Variable Kinetics of LF and bacteremia*. Bacteremia and LF represent the two sides of anthrax, infection and intoxication. Graphed over time in all 23 animals, they demonstrate the variability of progression and the differences in these two biomarkers (Fig 2A and 2B). Most animals appeared to have a triphasic kinetics of bacteremia and LF, with an initial rapid increase (phase-1), a plateau or decrease (phase-2), and a rapid increase leading to terminal stage (phase-3). The kinetic trends were similar between bacteremia and LF, but the changes were more dramatic for bacteremia, especially in phase-2, with declines for bacteremia and plateaus for LF.

*Disease progression*. Of 11 animals with shorter survival time (faster progressing) 7 animals had a triphasic kinetics of bacteremia and LF (Fig 2C and 2E), and 4 had monophasic kinetics, where bacteremia and LF increased directly from detection to the last sample (Fig 2D and 2F). The speed of progression meant that terminal plasma samples were not obtained for LF (or any toxin measurement) (Fig 2E and 2F). However, terminal measurements for bacteremia were available and provided evidence of the final rise (Fig 2C and 2D). The phase-1 rise of LF and bacteremia in animals with faster progression appeared to be to higher levels and the phase 2 plateaus were of shorter duration compared to animals with longer survival (Fig 2G and 2H).

In 11 non-survivors with longer survival (slower progressing), the phase-1 rise in LF and bacteremia were to lower levels followed by a plateau of variable length in phase-2 (Fig 2G and 2H). LF levels in phase-2 varied by less than one order of magnitude, from lowest at 4.8 ng/mL to highest at 41.5 ng/mL, across all animals and time points (boxed area). Conversely, phase-2 bacteremia varied over 4-orders of magnitude, from 1.76E+05 cfu/mL to low ‘+’ (Fig 2G). For other toxins, phase-2 for PA and LTx were less variable (similar to LF) and EF and ETx were more variable (similar to bacteremia) (S3 Fig). The final phase-3 rise in LF and bacteremia was rapid and to high levels for most animals.

**Fig 3:**
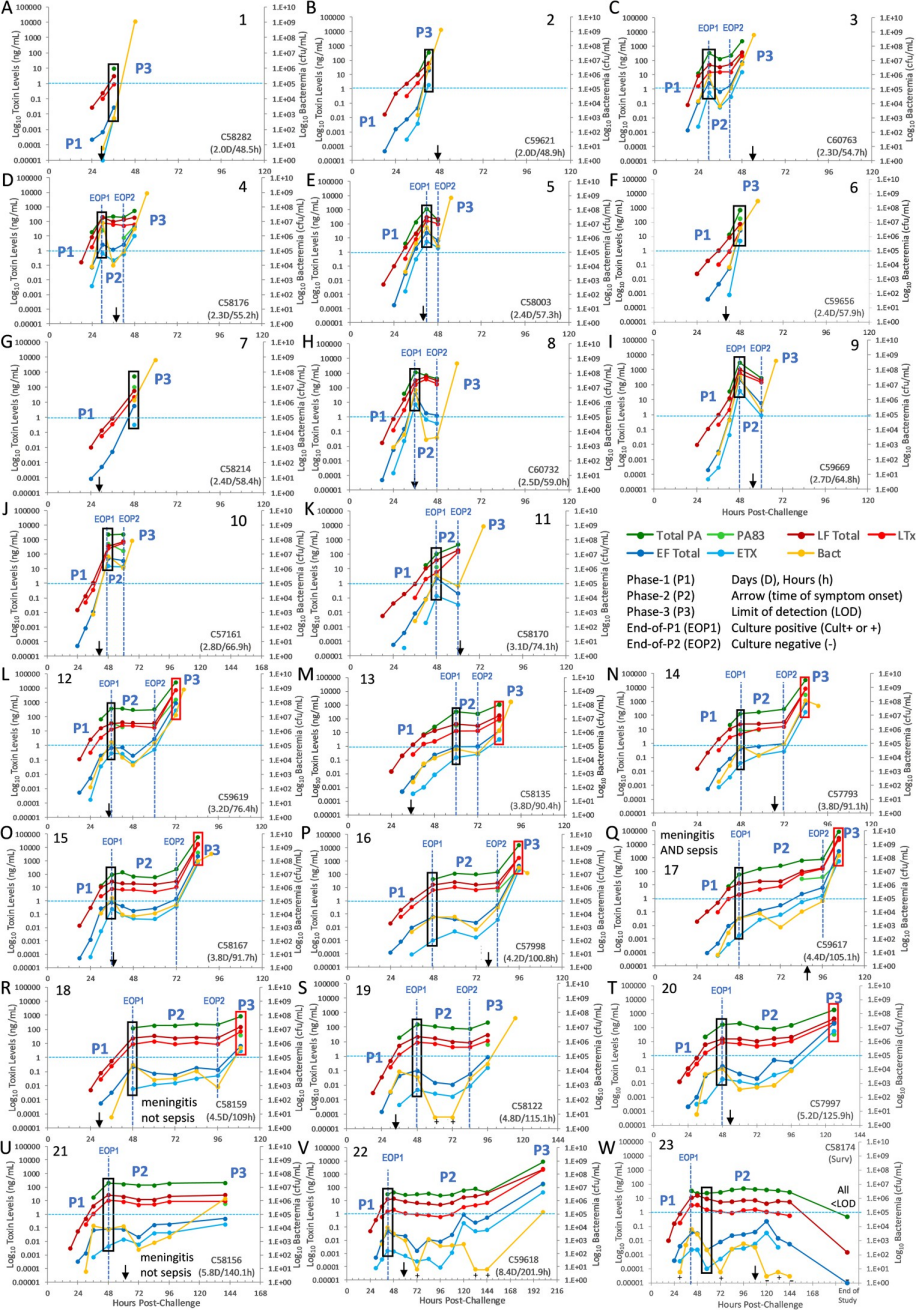
Anthrax toxins and bacteremia during inhalation anthrax in individual cynomolgus macaques. Total PA, PA83, LF, EF, LTx and ETx (ng/mL) and bacteremia (cfu/mL) in 11 animals with fast progression (A-K), 11 with slow progression (L-V), and the survivor (W). Phase-1 (P1), phase-2 (P2) and phase-3 (P3) are indicated when present. Blue vertical dashed lines indicate the point separating the phases; the end-of-P1 (EOP1) that ends the P1 rise and begins the P2 plateau and the end-of-P2 (EOP2) that ends P2 and begins P3. Black arrows indicate the time of symptom onset. Survival time-days (D), hours (h). Black rectangles at the end-of-phase-1 show how toxin levels are convergent (lower differences between highest toxin PA and lowest toxin ETx) in animals with shorter survival or divergent (higher differences between PA and ETx) in animals with longer survival. Red rectangles in phase-3 in animals with longer survival show convergent levels at terminal infection. Toxins (ng/mL) and bacteremia (cfu/mL) were graphed on a similar scale (10-orders of magnitude) for comparison of dynamic changes, orders of magnitude. Aqua horizontal dashed line at 1 ng/mL toxin concentration and 1.0E+05 cfu/mL bacteremia. Low positive culture (+) graphed at 6 cfu/mL and negative culture (-) at 3 cfu/mL. Less than the limit of detection (<LOD).

*All toxins and bacteremia*: *kinetics in individual animals*. A complete list of all toxin levels for each animal at each time point is included in a separate file (S2 Data File) and bacteremia is included in the S3 Data File. All toxins and bacteremia were graphed together for all 23 individual animals, numbered by survival time (Fig 3). Terminal plasma samples for toxin measurements were not available in animals 1 through 16, and 19. Terminal bacteremia showed the final kinetic trends to terminal in these animals.

Graphed together shows all toxins and bacteremia follow the same general kinetic profile. As seen for LF and bacteremia, the four animals with fast monophasic progression show an initial rise in all other toxins from phase-1 which continued with bacteremia to terminal stage (Fig 3A, 3B, 3F and 3G). Animal 1 only had toxins measured out to 36 hours post-challenge and therefore, levels weren’t as high as in the other three monophasic animals with toxins measured out to 42- and 48-hours. Terminal stage bacteremia reached high levels of 10^8^ to more than 10^9^ cfu/mL in all four. Based on the trends in other animals, the rise in bacteremia in the terminal sample of animal 1, suggests that toxins levels would have also risen to terminal. In the other seven animals with fast progression, six clearly had triphasic progression (animals 3–5, 8–10). The seventh, animal 11, had a mixed triphasic kinetics with phase-2 declines for EF, ETx and bacteremia, while PA, LF, and LTx levels did not plateau but continued to rise, albeit more slowly than in phase-1 (Fig 3K). As in animal 11, EF and ETx trended with bacteremia in all animals, with more dramatic declines in phase-2, whereas PA and LTx trended with LF, with plateaus in phase-2.

For the 11 non-survivors with longer survival (slow progression), kinetic profiles were triphasic in animals 12 through 20, and 22 (Fig 3). Animal 21 was the exception, with a long phase-2 plateau to terminal for all toxins, while only bacteremia started to rise in phase-3 (Fig 3V). Three phases were observed for all toxins, even in the six animals missing terminal toxemia. The changes in bacteremia in the final few hours showed a continued rise in four (Fig 3L, 3M, 3O and 3S), and a decline in two (Fig 3N and 3P).

*Potential relationship with survival time*. As shown above, LF and bacteremia appeared to rise to higher levels early in the 11 animals with fast progression. There also appeared to be an association between early levels of all toxins and subsequent survival time (Fig 3). Using the horizontal line at 1 ng/mL toxin concentration and 1.0E+05 cfu/mL bacteremia as a gauge, shows that the end-of-phase-1 toxins and bacteremia (black boxes), were higher in animals with shorter survival and trended lower in those that survived longer. Another trend was the magnitude and length of the phase-2 plateau, with higher concentrations and shorter duration in animals with short survival and lower concentrations and longer duration with longer survival. This suggests that early toxin levels may correlate with subsequent survival time. This association was explored below.

*Relationship of toxins to each other*. The detection of PA83 was sporadic, indicating that PA63 was the predominant form of total PA circulating. In addition, total PA, once positive, was the most abundant toxin, with PA>LF>LTx>EF>ETx (Fig 3). In animals with longer survival, the ranges of these toxins during phase-2 were more divergent, with 4- to 5-orders of magnitude between the concentrations of PA (highest) and ETx (lowest). Phase-2 toxins were the most divergent in animals 16 through 23 (black boxes). In phase-3, all toxins increased rapidly and converged (red boxes). In animals 2 through 11 with shorter survival, toxins reached higher levels and converged much earlier, approaching or at the end-of-phase-1. Therefore, lower toxin levels were associated with divergent toxemia (higher toxin ratios) and higher toxin levels with convergent toxemia (lower toxin ratios).

#### 2.4 Characterization of toxins, bacteremia, and toxin ratios stratified by phase as defined by toxemic profiles

*2.4.1 Phase-1*

*Toxemia in the early Incubation period*. The earliest time points at 18- and 24-hours post-challenge were collected before the earliest symptoms at 29 hours post-challenge. LF was positive in 12 animals by 18 hours post-challenge and in all 23 by 24 hours. Combined 18-to-24-hour median (interquartile range-IQR) LF was 0.025 (0.013–0.115) ng/mL (n = 35). EF was positive in 5 animals at 18 hours and 16 at 24 hours, at 0.00021 (0.0014–0.00007) ng/mL (n = 21). Other toxins and bacteremia were not detected at these early time points in sufficient numbers for assessment.

**Fig 4:**
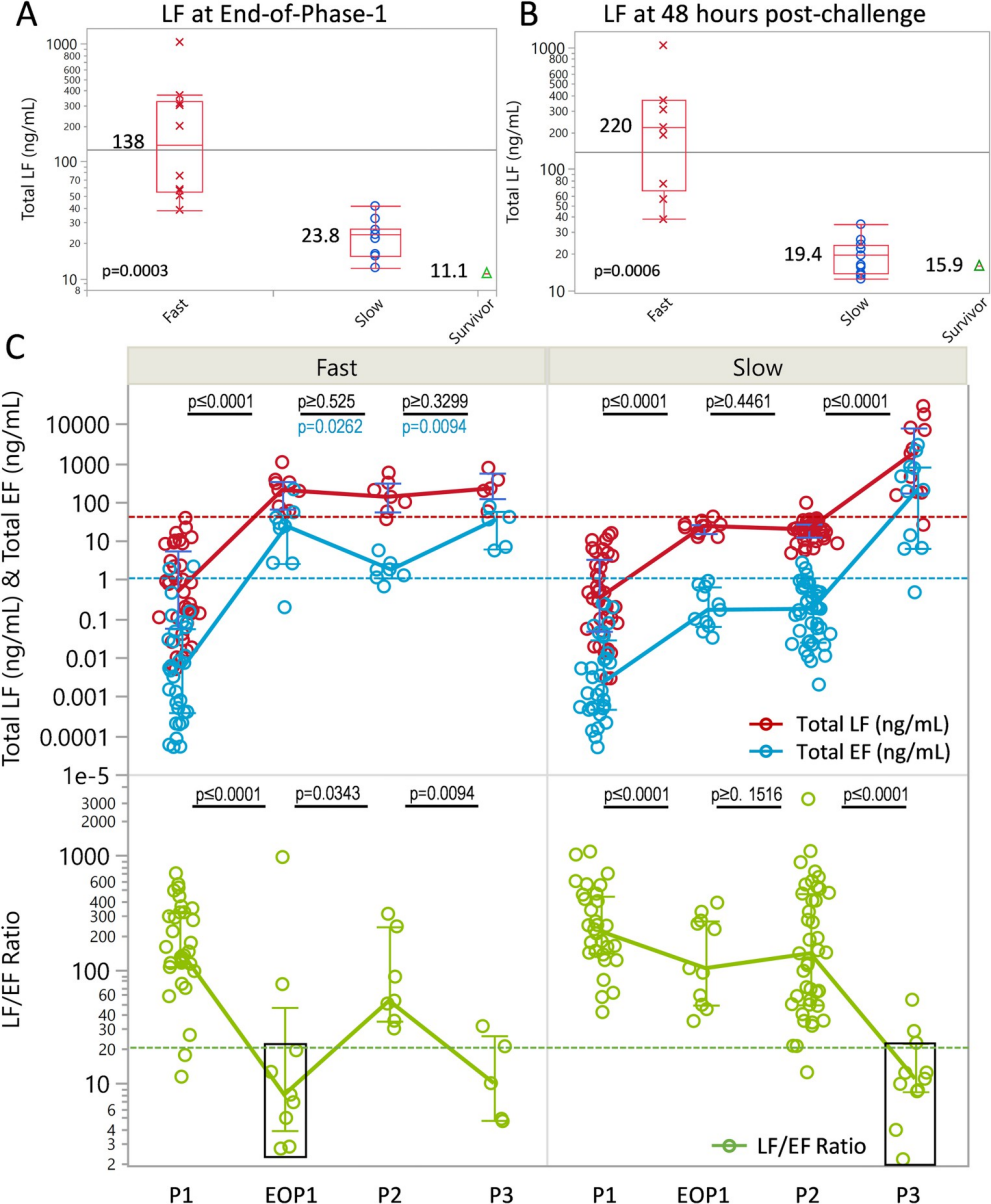
Stage-dependent biomarker levels. Lethal factor levels in animals with fast and slow progression, and survivor, at the end-of-phase-1 (A) and 48 hours (B). Box plot is for median and quartiles, error bars for minimum and maximums, excluding outliers, median levels and p-values included for Wilcoxon rank sum test. (C) LF, EF levels (upper panel), and LF/EF ratio (lower panel), during earliest detection in phase-1 (P1), at the end-of-phase-1 (EOP1), subsequent phase-2 (P2), and phase-3 (P3) for fast progression (left panel) and slow progression (right panel). Lines connect the medians and error bars give quartiles for each stage. Red dashed line represents the LF threshold and blue line the EF threshold for phase-2. P-values included for testing differences in toxin levels and toxin ratios between phases. Black font includes all parameters unless another color font is shown for a specific parameter. Black rectangle shows similarity of low LF/EF ratios at the EOP1 for fast progression and P3 for slow progression.

*End-of-phase-1*. The end-of-phase-1, was clearly observed in all but the four animals with monophasic progression (Fig 3). In these animals, the first rise ends at the terminal stage (phase-3). The timing to reach the end-of- phase-1 ranged from 30 to 60 hours post-challenge. By this time, all toxins and bacteremia, except for the unprocessed PA83, were positive in all animals. Characterization of the levels showed that all were significantly higher in the fast progression group than the slow progression group (Table 5 and Fig 4A). Toxin levels were even lower in the survivor. The differences in LF at the end-of-phase-1 were also observed at 48h (Fig 4A and 4B).

**Table 5:** Stage-dependent biomarker levels.

	End-of-Phase-1 (Beginning-of-Phase-2)			All of Phase-2				Phase-3 (≤12 hours from terminal)	
Parameter	Fast (n = 10)^10^	Slow (n = 11)^11^	Survived (n = 1)^1^	Fast (n = 7)^15^	Slow (n = 11)^52^	Survived (n = 1)^10^	Slow Phase-2 Threshold^#^ (n = 11)^52^	Fast (n = 9)^9^ 7.2(6.8–10.2)-hours	Slow (n = 10)^11^ 4.4(0–7.1)-hours
Total-PA (ng/mL)	620 (292–1447)	133 (54.2–200)^c^	33.9	326 (199–1189)	137 (67.4–205)^a^	34.6 (26.2–41.3)	342	509 (308–1477)	8547 (813–34638)^d^
Total LF (ng/mL)	138 (54.9–326)	23.8 (15.6–26.3)^b^	11.1	200 (98.1–366)	20.2 (13.9–26.1)^a^	6.7 (5.6–9.8)	35.8	200 (66.5–337)	1754 (171–7996)^f (ns)^
Lethal Toxin (ng/mL)	54.7 (19.6–188)	8.6 (4.6–10.3)^b^	3.25	139 (50.9–245)	7.38 4.51–10.3)^a^	1.3 (0.89–2.0)	18.9	97.8 (25.7–192)	1799 (92.9–7991)^e (ns)^
Total EF (ng/mL)	22.2 (2.7–42.8)	0.17 (0.064–0.66)^a^	0.040	2.68 (1.8–34.9)	0.172 (0.042–0.47)^a^	0.025 (0.0074–0.040)	1.10	20.7 (5.6–37.75)	201 (6.5–797)^e (ns)^
Edema Toxin (ng/mL)	3.4 (0.51–9.1)	0.0057 (0.0020–0.15)^b^	0.0023	0.663 (0.294–7.1)	0.035 (0.0048–0.095)^a^	0.0028 (0.0013–0.0046)	0.312	2 (0.62–11.9)	54.8 (2.9–272)^d^
Bact (cfu/mL)	4.8E+06 (1.2E+06–7.6E+06)	1.1E+04 (5.6E+03–6.2E+04^a^	6.4E+03	2.6E+05 (6.3E+04–7.2E+06)	4.9E+03 (7.1E+02–1.4E+04)^a^	2.4E+02 (5.3E+00–1.2E+03)	1.5E+05	1.3E+06 (2.2E+05–3.4E+06)	6.7E+06 (3.9E+05–9.7E+07)^f (ns)^
								0h-Term (n = 11) 6.3E+08 (4.0E+08–8.8E+08)	0h-Term (n = 10) 4.4E+07 (1.2E+06–2.1E+08)^c^
PA/LF Ratio	4.8 (2.9–7.1)	5.6 (3.4–7.9)^g (ns)^	3.1	2.9 (1.4–3.8)	7.0 (4.4–8.8)^a^	5.3 (2.9–6.9)	2.5	3.1 (1.4–7.6)	4.3 (3.4–6.3)^f (ns)^
LF/LTx Ratio	2.4 (1.8–2.9)	2.7 (2.5–5.2)^f (ns)^	3.4	1.8 (2.4–1.4)	2.6 (2.3–3.8)^b^	5.0 (4.4–6.6)	1.7	2.2 (1.5–2.7)	1.20 (1.0–1.8)^d^
LF/EF Ratio	9.0 (4.4–18.2)	103 (49.2–270)^b^	278	31.9 (12.7–74.6)	127 (50.8–400)^b^	350 (234–761)	21.4	10.1 (3.7–33.8)	11.0 (8.6–22.8)^g (ns)^
EF/ETx Ratio	5.4 (4.2–12.3)	16.0 (6.0–30.7)^e (ns)^	17.4	4.5 (3.6–5.7)	5.5 (3.7–11.7)^f (ns)^	9.9 (6.8–16.7)	2.7	4.8 (3.5–8.8)	3.7 (2.6–4.3)^e (ns)^
PA/ETx Ratio	190 (148–615)	19,733 (2144–31,667)^c^	14739	323 (167–986)	4565 (1383–23,958)^a^	13,467 (8967–35804)	506	171 (118–733)	198 (86.6–338)^g (ns)^

Triphasic kinetics of toxemia is defined by LF levels that increase to the end-of-phase-1, a plateau/decline in phase-2, and a final increase in phase-3. Median (lower quartile–upper quartile) (determined in JMP) are given for fast (n = 11) and slow (n = 11) progression groups, and the animal that survived (animal 23, C58174) at the end-of-phase-1, all points making up phase-2, and phase-3 (available measurements within 12 hours of terminal). The survivor did not have phase-3. Toxin ratios were included for PA/LF, LF/EF, LF/LTx, EF/ETx and PA/ETx (highest- to lowest-level toxin). PA83 excluded. Of 23 animals, all but one animal (fast) had an end-of-phase-1 sampleNumbers of animals included in each measurement (n) with the superscripts for the number of measurements e.g. (n = 10)^10^ included for each parameter and phase of infection. All points per animal for phase-2 with slow progression (n = 11)^52^, except for ETx (n = 11)^51^. The limits of phase-2 for slow progression, the upper 95^th^ percentile (%) for toxins and bacteremia and lower 95^th^ percentile for toxin ratios, was included as a threshold for exiting phase-2 (and entry to phase-3). P-values are from Wilcoxon Rank Sum Test in JMP, for differences between fast vs slow progression. Letter superscripts indicate p-values where, a (p≤0.0001), b (p≤0.001), c (p≤0.01), d (p≤0.05), e (p≤0.1), f (p≤0.5), g (p≤1.0). Alpha level set at 0.05 indicating significant differences were observed for superscripts a-d, but e-g were not significant (ns).

Higher end-of-phase-1 toxemia in fast progression was associated with lower toxin ratios and lower toxemia in slow progression with higher ratios (Table 5). These differences were significant for LF/EF and PA/ETx. LF/LTx and EF/ETx also trended lower for fast progression but differences were not significant. PA/LF did not fit the trend and was similar for fast and slow progression at the end-of-phase-1. Individual animal measurements at the end-of phase-1 for all toxins (including PA83), toxin ratios, and bacteremia, are included (S1 Table).

*2.4.2 Phase-2*

The end-of-phase-1 is also the beginning of phase-2, which follows with a plateau/decline. Therefore, toxin levels at the end-of-phase-1 largely remained unchanged during phase-2. This was more evident for slow progression than for fast progression which declined more than plateaued in phase-2 (Table 5 and Fig 4C). Phase-2 levels of toxins and bacteremia were significantly higher and toxin ratios lower (except for EF/ETx) for the 7 animals with phase-2 in fast progression, compared to the 11 animals with slow progression (Table 5). In the one survivor, phase-2 toxin levels and bacteremia were even lower and toxin ratios higher (except for PA/LF), than in animals with slow progression.

*Phase-2 critical thresholds for entry to phase-3*. In slow progression, phase-2 LF levels varied less than one order of magnitude for the entire phase-2 period in all 11 animals (Fig 2H). This narrow range of LF represents a period where relatively small increases in LF may move infection to the final stage 3. Median, interquartile ranges, and upper and lower 95% confidence intervals characterized this period (Table 5). The upper 95% interval represents an upper confidence limit. Toxin levels above this limit fail the criteria for belonging in phase-2. Therefore, the upper limit represents a potential threshold above which an animal has exited phase-2 and entered phase-3. For toxin ratios, the lower 95% confidence limit applies. The thresholds for all toxins and ratios are included (Table 5).

*2.4.3 Phase-3*

Phase-3, as defined by toxin kinetics, is the rapid rise in toxin levels during the final hours of terminal infection as seen for each animal (Fig 3). Its’ characterization requires consideration of two main points. One point is the speed of this stage which resulted in relatively few samples collected for toxin measurements at terminal times. Therefore, we chose to include any measurements for time points collected within 12 hours of terminal time. The second point is that for animals with fast progression, the kinetic features are compressed within the last 12-to-24 hours (Fig 3). For these animals, points within 12 hours of terminal may include those at the end-of-phase-1 or phase-2. Bacteremia, which did have terminal measurements, was characterized two ways; 1) with the same time points as available for toxins and 2) with the final terminal time points (0 hours before terminal).

With the limitations described above, phase-3 toxemia was compared between animals with fast and slow progression (Table 5 and Fig 4C). Phase-3 samples for fast progression were collected at median (interquartile range) of 7.2 (6.8–10.2) hours before terminal, which was earlier than those for slow progression at 4.4 (0–7.1) hours before terminal. Phase-3 toxin levels and bacteremia were higher for animals with slow progression than fast progression, but the differences were not significant except for PA and ETx (Table 5). Of the toxin ratios only LF/LTx was significantly lower in slow progression than fast progression and approached 1:1 in slow progression, indicating that LTx nearly equals total LF with 100% existing in complex with PA63. With rapid changes during this stage, the differences between groups may simply be an artifact of the differences in collection times. Bacteremia at the final terminal collection times, zero hours from terminal, was significantly higher in animals with fast progression than in those with slow progression (Table 5).

Overall, from first detection to the terminal stage, all toxins and bacteremia increase whereas all toxin ratios (except PA/LF) decline. These trends, increasing toxemia and declining ratios through the phases may be used to define early and intermediate stages, with the phase-2 thresholds indicating the transition to late-stage anthrax.

*Comprehensive staging with anthrax progression*. Fig 4C summarizes four aspects of these three phases, early phase-1, the end-of-phase-1, subsequent phase-2, and phase-3, for LF, EF, and LF/EF ratios. In early phase-1, LF and EF levels, and LF/EF ratios, were similar for fast and slow progression, both with low levels and high ratios. From early phase-1 to the end-of-phase-1, there were significant increases in LF and EF levels and decreases in LF/EF ratios in both groups, but to higher levels and lower ratios in animals with fast progression. At the end-of-phase-1, most LF and EF points were above the phase-2 thresholds in animals with fast progression and at or below the thresholds in animals with slow progression. LF/EF ratios at the end-of-phase-1 in fast progression were low and similar to those at phase-3 in slow progression. For fast progression, LF levels did not change from the end-of-phase-1 through remaining phases, whereas EF levels declined significantly from the end-of-phase-1 to phase-2, then increased from phase-2 to phase-3, effecting an inversion of the LF/EF ratio. For slow progression, LF and EF did not change from the end-of-phase-1 through phase-2, whereas LF and EF levels increased from phase-2 to phase-3, and LF/EF ratios declined.

#### 2.5 Relationship of toxemia and time

*2.5.1 Time to reach phase-2 threshold and time from threshold to terminal*

**Fig 5:**
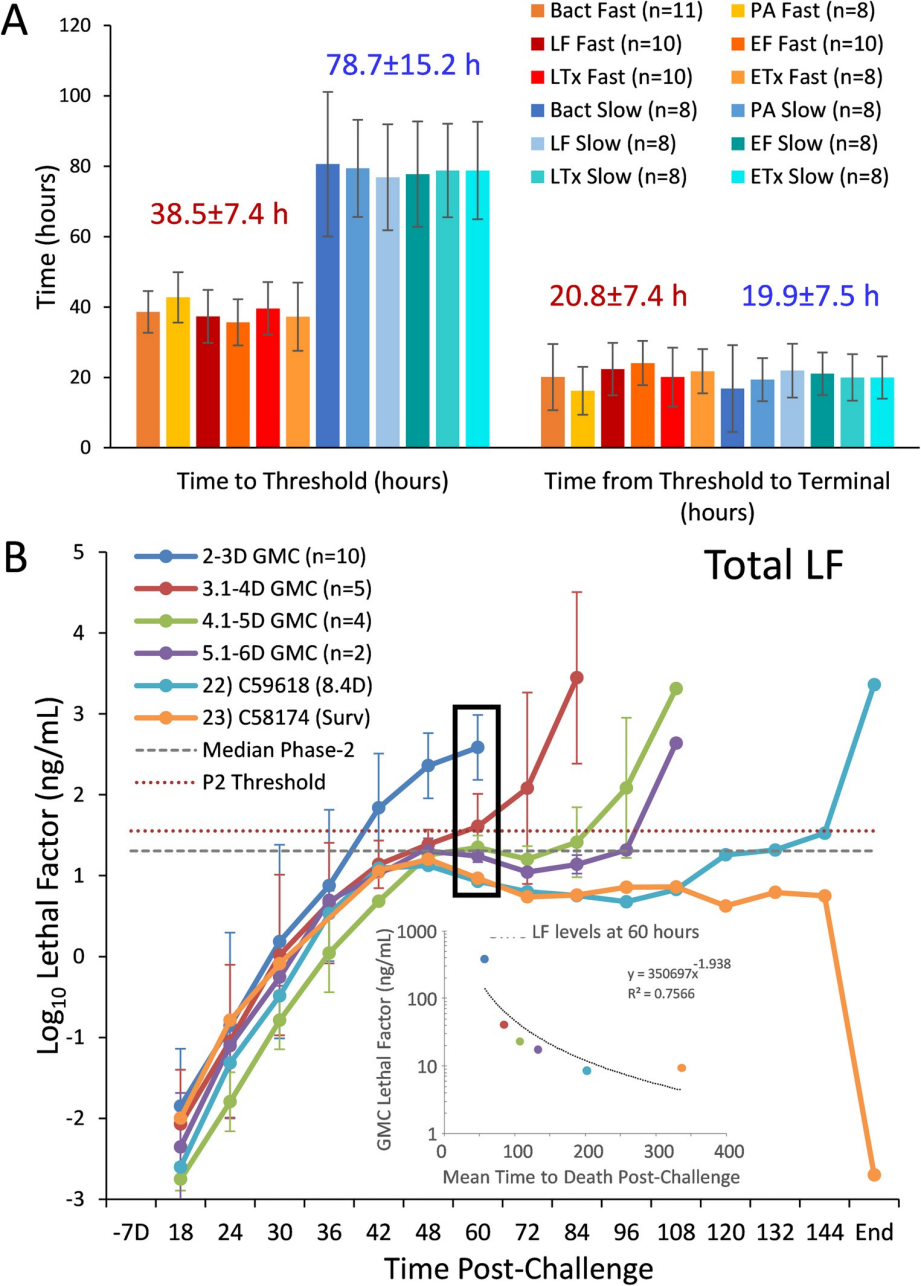
Relationship of toxemia and time. (A) Time in hours (h) from spore exposure to reach the phase-2 threshold for exiting phase-2 and entry to phase-3 (Table 5) and time from threshold to terminal. The time to reach the threshold was calculated for individual animals for all toxins and bacteremia and means and standard deviation (SD) (error bars) for the number (n) animals was determined for fast and slow progression. The overall mean±SD for all toxins and bacteremia combined for fast and slow progression is included above the bars. (B) Relationship between LF levels and survival time (days). Mean log_10_ concentrations and standard deviations as error bars for total LF over the time post-challenge (hours-h) for animals that survived 1.9–3.0 days (n = 10), 3.1 to 4.0 days (n = 5), 4.1 to 5 days (n = 4) and 5.1 to 6 days (n = 2), and for one animal at 8.4 days and survivor to end-of-study (End). Final time points for 22) C59618 at 8.4 days and 23) C58174 the survivor at end-of-study (14 days) were included together (End). Horizontal lines shown for median phase-2 LF (dashed grey) and LF phase-2 threshold (red dotted line). The black rectangle for LF at 60 h for mean log_10_ LF graphed vs mean subsequent survival time (60-h to terminal) (inset), fitted with a power regression.

The time to reach the toxin thresholds was calculated for each animal for all toxins and bacteremia. The survivor never reached the phase-2 mean so was excluded. Some animals were excluded for missing relevant samples. On average, the animals with fast progression reached the thresholds in about half the time (38.5±7.4 hours) of the animals with longer survival times (78.7±15.2 hours) (Fig 5A). After reaching the threshold, the time to terminal was remarkably similar for all animals, with 20.8±7.4 hours for fast and 19.9±7.5 hours for slow progression. These calculations were consistent for all biomarkers. This suggests that the time to reach critical toxin thresholds is variable but once it is exceeded, the course is rapid and similar.

*2.5.2 Relationship between LF levels and survival time*

Animals with faster progression reached critical thresholds of toxin levels earlier and died sooner than those with slower progression. However, the time-to-death for animals with slow progression varied from 3.8- to 8.4-days. The relationship between LF levels and the time-dependent interval to death was demonstrated by grouping animals by survival time (Fig 5B). LF levels in animals surviving 2.0- to 3.0-days (n = 10) reached the LF threshold first. Those surviving 3.1 to 4.0 days (n = 5) reached the threshold second and the pattern continued with fewer animals and longer survival times reaching threshold levels later. At 60 hours there was a negative relationship between LF levels and survival time. The pattern observed was strongest for LF but was present for all toxins and bacteremia (S4 Fig). Grouping by survival time showed the relationship between toxin levels and survival time and the reliability with which exceeding the phase-2 threshold was followed by a short interval to death.

The toxemia profiles for two animals with long progression, animal 23 that survived to the end of the study and animal 22 that survived 8.4 days, were similar as shown for LF out to 108 hours, both below the median for phase-2 (Fig 5B). By 108- to 120-hours toxins diverged, increasing in the non-survivor (S4 Fig). In the final sample, all toxins and bacteremia were high for the non-survivor and non-detectable for the survivor.

### 3. Early hematology associated with time and progression

Samples were collected for hematological parameters through 72 hours in challenge group-1 and 144 hours in challenge group-2. Only two of 11 animals with fast progression had measurements after 48 hours. This prohibited comparison of hematology between survival groups beyond 48h. Most animals had measurements for 7 days pre-challenge (day -7), and 24-, 36- and 48-hours post-challenge. Therefore, these four time points were assessed for both changes with time and differences between fast (high early toxemia) and slow (low early toxemia) progression groups and the survivor. All hematology measurements, including those after 48 hours, are included in the S4 Data File.

**Fig 6:**
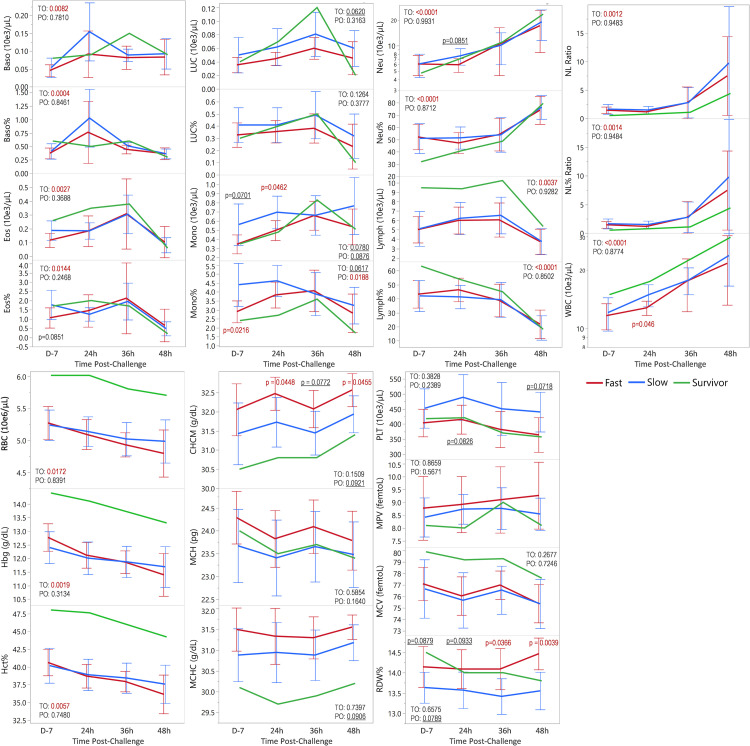
Early hematology during inhalation anthrax. Means and 95% confidence intervals graphed from day -7 (7 days pre-challenge) to 48 hours(h) post-challenge for fast progression (n = 11, n = 9 at 48 h), slow progression (n = 11), and survivor (n = 1). Differences between fast and slow progression at individual time points were determined by one-way ANOVA. P-values ≤0.10 were included in black font and p-values ≤0.05 in red font. All means, confidence intervals and p-values are included in Table 6. JMP fit model was used to test the effect of hematology with time (day -7, 24 h, 36 h and 48 h) and progression (fast vs slow). P-value for time overall (TO) and progression overall (PO) is given. Basophils (Baso), eosinophils (Eos) large unstained cells (LUC), monocytes (Mono), neutrophils (Neu), lymphocytes (Lymph), neutrophil lymphocyte ratio (NL Ratio), white blood cells (WBC), red blood cells (RBC), hemoglobin (Hgb), hematocrit (Hct), corpuscular hemoglobin concentration mean (CHCM), mean corpuscular hemoglobin (MCH), mean corpuscular hemoglobin concentration (MCHC), platelets (PLT), mean platelet volume (MPV), mean corpuscular volume (MCV), red cell distribution width (RDW).

Table 6 includes means for fast progression, slow progression, and values for the survivor, for individual time points for all parameters. These are graphed for visualization of trends for time overall and progression overall (Fig 6).

**Table 6:** Early hematology in cynomolgus macaques with inhalation anthrax.

Parameter	Day -7				24 hours				36 hours				48 hours			
	Fast (n = 11)	Slow (n = 11)	Surv	p-value	Fast (n = 11)	Slow (n = 11)	Surv	p-value	Fast (n = 11)	Slow (n = 11)	Surv	p-value	Fast (n = 9)	Slow (n = 11)	Surv	p-value
Baso (10e3/μL)	0.045 (0.024–0.067)	0.054 (0.032–0.075)	0.080	0.576	0.092 (0.023–0.161)	0.155 (0.085–0.224)	0.090	0.1965	0.082 (0.057–0.106)	0.089 (0.065–0.114)	0.15	0.6657	0.083 (0.039–0.128)	0.093 (0.052–0.133)	0.09	0.7476
Baso%	0.373 (0.258–0.488)	0.409 (0.294–0.524)	0.60	0.647	0.764 (0.243–1.28)	1.03 (0.507–1.55)	0.50	0.4639	0.446 (0.377–0.514)	0.509 (0.441–0.577)	0.60	0.185	0.367 (0.270–0.464)	0.364 (0.276–0.451)	0.30	0.9617
Eos (10e3/μL)	0.117 (0.0595–0.175)	0.188 (0.130–0.246)	0.260	0.0851	0.184 (0.100–0.267)	0.186 (0.102–0.269)	0.35	0.9747	0.309 (0.116–0.502)	0.306 (0.114–0.499)	0.38	0.9835	0.104 (0.023–0.186)	0.082 (0.008–0.155)	0.06	0.6689
Eos%	1.06 (0.424–1.70)	1.77 (1.13–2.41)	1.70	0.118	1.45 (0.817–2.07)	1.25 (0.626–1.88)	2.00	0.659	2.13 (0.693–3.56)	1.95 (0.521–3.39)	1.7	0.8608	0.656 (0.042–1.27)	0.473 (-0.082–1.03)	0.20	0.6479
LUC (10e3/μL)	0.035 (0.017–0.054)	0.050 (0.031–0.069)	0.040	0.270	0.045 (0.025–0.064)	0.062 (0.043–0.081)	0.070	0.1995	0.060 (0.036–0.084)	0.081 (0.057–0.105)	0.12	0.2132	0.046 (0.020–0.071)	0.060 (0.037–0.083)	0.02	0.3847
LUC%	0.327 (0.212–0.443)	0.409 (0.294–0.524)	0.30	0.307	0.355 (0.242–0.467)	0.409 (0.297–0.521)	0.40	0.4816	0.382 (0.231–0.532)	0.491 (0.340–0.641)	0.5	0.2978	0.233 (0.053–0.414)	0.318 (0.155–0.481)	0.10	0.4735
Lymph (10e3/μL)	5.01 (3.48–6.54)	5.11 (3.57–6.64)	9.46	0.930	5.99 (4.52–7.47)	6.22 (4.74–7.69)	9.35	0.8239	6.05 (4.29–7.81)	6.53 (4.77–8.29)	10.2	0.6928	3.75 (2.41–5.09)	3.75 (2.54–4.97)	5.35	0.9948
Lymph%	43.2 (33.5–53.0)	42.0 (32.2–51.8)	63.3	0.857	46.4 (38.6–54.2)	41.3 (33.5–49.2)	53.4	0.3501	38.8 (27.6–50.1)	39.3 (28.0–50.6)	45	0.9532	21.9 (12.8–31.0)	19.2 (10.9–27.5)	18.1	0.6521
Mono (10e3/μL)	0.346 (0.181–0.512)	0.561 (0.396–0.726)	0.350	0.0701	0.497 (0.359–0.635)	0.696 (0.558–0.835)	0.480	**0.0462**	0.660 (0.483–0.837)	0.663 (0.486–0.840)	0.83	0.9821	0.538 (0.268–0.807)	0.766 (0.523–1.01)	0.51	0.2028
Mono%	2.90 (2.00–3.79)	4.41 (3.52–5.30)	2.40	**0.0216**	3.84 (3.09–4.59)	4.63 (3.88–5.38)	2.70	0.1352	4.07 (2.97–5.18)	3.89 (2.78–5.00)	3.6	0.8112	2.82 (1.79–3.85)	3.25 (2.31–4.18)	1.7	0.5299
Neu (10e3/μL)	6.09 (4.45–7.73)	6.11 (4.47–7.76)	4.76	0.982	5.97 (4.65–7.29)	7.59 (6.27–8.91)	7.15	0.0851	10.4 (5.62–15.2)	10.1 (5.33–14.9)	11.0	0.9281	17.2 (9.29–25.1)	19.1 (11.9–26.2)	23.53	0.7187
Neu%	52.1 (41.6–62.6)	51.0 (40.4–61.5)	31.8	0.874	47.2 (39.0–55.5)	51.4 (43.1–59.6)	40.9	0.4651	54.1 (41.3–67.0)	53.9 (41.0–66.7)	48.6	0.9762	74.0 (63.8–84.3)	76.4 (67.1–85.7)	79.6	0.7216
NL Ratio	1.46 (0.769–2.14)	1.63 (0.947–2.32)	0.503	0.707	1.17 (0.663–1.67)	1.49 (0.986–1.99)	0.765	0.3542	2.76 (0.190–5.33)	2.80 (0.231–5.37)	1.08	0.9813	7.48 (-1.32–16.3)	9.74 (1.78–17.7)	4.398	0.6937
NL% Ratio	1.45 (0.767–2.14)	1.63 (0.947–2.32)	0.502	0.704	1.17 (0.663–1.67)	1.49 (0.986–1.99)	0.766	0.3550	2.76 (0.198–5.31)	2.79 (0.236–5.35)	1.08	0.9828	7.48 (-1.44–16.4)	9.81 (1.75–17.9)	4.398	0.6883
PLT (10e3/μL)	404 (351–457)	452 (399–505)	418	0.192	415 (356–475)	489 (430–549)	421	0.0826	382 (311–453)	451 (380–522)	370	0.1687	364 (302–426)	440 (384–497)	357	0.0718
MPV (femtoL)	8.77 (7.81–9.74)	8.42 (7.46–9.38)	8.10	0.593	8.92 (8.10–9.74)	8.74 (7.92–9.55)	8.00	0.746	9.1 (8.09–10.1)	8.76 (7.76–9.77)	9.0	0.6273	9.27 (8.34–10.2)	8.55 (7.71–9.38)	8.1	0.2407
WBC (10e3/μL)	11.6 (9.69–13.6)	12.1 (10.1–14.0)	15.0	0.753	12.8 (11.3–14.3)	14.9 (13.4–16.4)	17.5	**0.046**	17.6 (13.6–21.6)	17.8 (13.8–21.8)	22.7	0.9387	21.7 (14.1–29.3)	23.8 (16.9–30.7)	29.56	0.6736
CHCM (g/dL)	32.1 (31.4–32.8)	31.4 (30.7–32.1)	30.5	0.199	32.5 (32.0–33.0)	31.7 (31.2–32.2)	30.8	**0.0448**	32.1 (31.6–32.6)	31.4 (30.9–31.9)	30.8	0.0772	32.6 (32.1–33.0)	31.9 (31.5–32.4)	31.4	**0.0455**
Hbg (g/dL)	12.8 (12.3–13.3)	12.4 (11.9–12.9)	14.4	0.294	12.1 (11.6–12.6)	12.0 (11.5–12.5)	14.1	0.753	11.9 (11.4–12.3)	11.9 (11.4–12.3)	13.7	0.9775	11.4 (10.7–12.2)	11.7 (11.0–12.4)	13.3	0.5534
Hct%	40.6 (38.6–42.7)	40.2 (38.1–42.2)	48.0	0.755	38.7 (36.9–40.5)	38.9 (37.1–40.7)	47.6	0.8791	37.9 (36.2–39.6)	38.4 (36.7–40.1)	45.9	0.6802	36.1 (33.5–38.8)	37.6 (35.1–40.0)	44.2	0.4188
MCH (pg)	24.3 (23.6–24.9)	23.7 (23.0–24.3)	24.0	0.178	23.8 (23.1–24.5)	23.4 (22.7–24.1)	23.5	0.3790	24.1 (23.5–24.7)	23.7 (23.0–24.3)	23.7	0.3269	23.8 (23.1–24.5)	23.5 (22.9–24.1)	23.4	0.4909
MCHC (g/dL)	31.5 (31.0–32.0)	30.9 (30.3–31.4)	30.1	0.109	31.3 (30.7–32.0)	30.9 (30.3–31.6)	29.7	0.3892	31.3 (30.8–31.8)	30.9 (30.4–31.4)	29.9	0.2525	31.6 (31.2–31.9)	31.2 (30.8–31.5)	30.2	0.1435
MCV (femtoL)	77.1 (75.2–79.1)	76.7 (74.7–78.6)	79.9	0.744	76.1 (74.1–78.0)	75.7 (73.7–77.6)	79.2	0.7752	77 (75.4–78.6)	76.6 (75.0–78.2)	79.3	0.6938	75.4 (73.4–77.3)	75.4 (73.6–77.1)	77.6	0.9842
RBC (10e6/μL)	5.27 (5.04–5.50)	5.24 (5.01–5.47)	6.01	0.833	5.09 (4.88–5.31)	5.14 (4.92–5.35)	6.01	0.7655	4.93 (4.72–5.14)	5.02 (4.81–5.23)	5.8	0.5467	4.80 (4.45–5.14)	4.98 (4.67–5.30)	5.7	0.4047
RDW%	14.1 (13.7–14.6)	13.6 (13.2–14.1)	14.5	0.0879	14.1 (13.7–14.5)	13.6 (13.1–14.0)	14.0	0.0933	14.1 (13.6–14.5)	13.4 (13.0–13.9)	14.0	**0.0366**	14.5 (14.0–14.9)	13.6 (13.2–13.9)	13.8	**0.0039**

Means and 95% confidence intervals from Oneway ANOVA, assuming equal variances, for 11 animals with fast progression, 11 animals with slow progression, and one animal that survived (surv) to the end-of-study, on day -7 (7 days pre-challenge), 24-, 36-, and 48-hours post-challenge (determined in JMP). P-values ≤0.10 are underlined and p-values ≤0.05 are in bold font. Basophils (Baso), eosinophils (Eos), large unstained cells (LUC), monocytes (Mono), neutrophils (Neu), lymphocytes (Lymph), neutrophil lymphocyte ratio (NL Ratio), white blood cells (WBC), red blood cells (RBC), hemoglobin (Hgb), hematocrit (Hct), corpuscular hemoglobin concentration mean (CHCM), mean corpuscular hemoglobin (MCH), mean corpuscular hemoglobin concentration (MCHC), platelets (PLT), mean platelet volume (MPV), mean corpuscular volume (MCV), red cell distribution width (RDW).

*Changes in early hematology with time*. For time overall (TO), there were significant increases in neutrophils, neutrophil%, neutrophil/lymphocyte (NL) ratio, NL% ratio, and white blood cells (WBC) (Fig 6). There were also significant changes over time for basophils, basophil%, eosinophils, and eosinophil%, with increases at 24 hours and/or 36 hours, and subsequent declines. Lymphocytes and lymphocyte% declined from 36 hours to 48 hours. Red blood cells (RBC), hemoglobin, and hematocrit declined continuously from pre-challenge through 48 hours. These changes were observed for both fast and slow progression. Other parameters did not change significantly over time.

*Differences in early hematology with progression*. Considering the speed with which some animals reach the late stage, differences at any one or more early time points may be related to progression (Table 6). For progression overall (PO), the mean for monocyte% was significantly higher in slow progression than fast progression (p = 0.0188) (Fig 6). For individual time points, means for monocyte% on day -7, and monocyte count and white blood cells at 24 hours, were significantly higher in slow progression. Platelets were also higher in slow progression, approaching significance at 24 hours and 48 hours. In contrast, progression overall means for red cell distribution width (RDW), corpuscular hemoglobin concentration mean (CHCM), and mean corpuscular hemoglobin concentration (MCHC) were higher in fast progression than slow, approaching significance (p ≤ 0.0921). At individual time points means for RDW were significantly higher for fast progression at 36 and 48 hours and CHCM at 24 and 48 hours (p ≤ 0.0455) with other time points approaching significance (p<0.1). Compared to all non-surviving animals, with both fast and slow progression, the survivor had higher lymphocytes, among the highest WBC count, exceptionally high RBC, hemoglobin, and hematocrit, higher MCV, and lower MCHC and CHCM. Comparing the survivor and the animal surviving to 8.4 days, at 132 hours, the RBC count in the survivor was within normal limits (5.0 to 7.6 e^6^/μL) at 5.24 e^6^/μL but was below normal in the non-survivor (3.85 e^6^/μL) (S4 Data File).

Collectively, the results show that higher levels of innate immune parameters (such as monocytes and WBC) at early time points were associated with slower progression and lower levels with fast progression. Conversely, higher levels of parameters associated with compromised red blood cell fitness, or hemolytic anemia (CHCM, MCHC and RDW) favored fast progression and lower ones slow.

### 4. Pathology

Assessment of terminal stage pathology can provide evidence of the impact of the toxemia and bacteremia measured during infection. Animals underwent complete necropsy, and gross and microscopic observations were recorded. Microscopic observations were assigned a semi-quantitative severity score as follows: ‘minimal’ for a minimally detectable lesion (Grade 1), ‘mild’ for an easily visualized lesion (Grade 2), ‘moderate’ for a lesion affecting larger areas of the tissue (Grade 3), and ‘marked’ for lesions affecting maximum areas of the tissue (Grade 4). Microscopic observations attributed to anthrax in this study included the presence of bacteria and pathologic changes, such as fibrin, hemorrhage, necrosis, inflammation, and edema when present (Table 7).

**Table 7:** Microscopic and gross pathology summary.

		Microscopic Tissue Bacteria																									
		Fast Progression (n=11)													Slow Progression (n=11)												
	Animal # → Organ↓	1	2	3	4	5	6	7	8	9	10	11	% Affected	Mean±SD Fast	12	13	14	15	16	17	18	19	20	21	22	% Affected	Mean±SD Slow
	Brain	1	2	1	1	1	1	1	1	0	1	0	**81.8**	0.91±0.54	1	0	0	0	1	2	2	0	0	1	0	**45.5**	0.64±0.81
	Kidney	1	2	2	2	1	1	1	1	2	2	2	**100**	1.5±0.52	2	2	1	2	1	1	0	1	1	0	1	**81.8**	1.1±0.7
	Liver	0	2	2	2	2	2	1	1	1	2	2	**91.7**	1.5±0.69	2	1	2	1	2	2	0	2	1	0	2	**81.8**	1.4±0.81
	Lung	1	2	2	1	2	2	2	2	2	0	3	**91.7**	**1.7±0.79**	2	3	1	3	2	3	0	2	1	0	2	**81.8**	**1.7±1.1**
	BLN	1	2	2	1	1	2	1	1	1	1	1	**100**	1.3±0.47	2	1	1	2	1	2	0	1	1	0	1	**81.8**	1.1±0.7
	MnLN	0	0	1	1	1	2	0	1	1	1	0	**66.7**	0.7±0.65	2	2	1	1	0	2	1	1	1	0	1	**81.8**	1.1±0.7
	MsLN	0	0	1	1	1	1	1	1	2	2	0	**75**	0.9±0.7	1	1	1	2	0	1	1	1	1	1	2	**90.9**	1.1±0.54
	Spleen	2	4	3	4	3	3	2	3	4	2	4	**100**	**3.1±0.83**	3	4	4	4	2	4	1	1	1	0	2	**90.9**	**2.4±1.5**
	**Total Bact**	6	14	14	13	12	14	9	11	13	11	12		11.7±2.5	15	14	11	15	9	17	5	9	7	2	11		10.5±4.6
Organ	Pathology	Microscopic and Gross Pathology																									
Brain	Fibrin	0	0	0	0	0	0	0	0	0	0	0	**0**	0	0	0	0	0	0	0	2	0	0	1	0	**18.2**	0.27±0.65
	Inflammation	0	0	0	0	0	0	0	0	0	0	0	**0**	0	0	0	0	0	0	2	2	0	0	3	0	**27.3**	0.64±1.12
	Hemorrhage	0	0	0	0	0	0	0	0	0	1	0	**9.1**	0.09±0.3	0	0	0	0	0	2	2	0	0	2	0	**27.3**	0.55±0.93
Kidney	Inflammation	0	0	0	0	0	0	0	0	0	1	0	9.1	0.09±0.3	0	0	0	0	0	0	1	0	0	0	0	9.1	0.09±0.3
	Necrosis	0	0	0	0	0	1	0	0	0	0	1	18.2	0.18±0.4	1	0	0	1	0	1	0	0	1	0	0	36.4	0.36±0.5
Liver	Inflammation	0	0	0	0	0	0	0	0	0	0	0	**0**	0	0	0	0	0	0	1	0	0	0	2	2	**27.3**	0.45±0.82
	Hemorrhage	0	0	0	0	0	0	0	0	0	0	0	0	0	0	0	0	0	0	0	0	0	0	2	0	9.1	0.18±0.6
	Necrosis	0	0	0	0	0	0	0	0	0	0	0	**0**	0	0	0	0	1	1	1	0	1	1	2	1	**63.6**	0.73±0.65
	Leukocytosis	1	1	2	2	1	1	2	0	2	2	1	**90.9**	**1.36±0.67**	1	1	1	2	1	2	1	1	2	2	2	**100**	**1.45±0.52**
Lung	Fibrin	0	0	0	0	0	0	0	0	0	0	0	**0**	0	0	0	0	0	0	0	1	0	1	1	1	**36.4**	0.36±0.5
	Inflammation	1	0	1	1	1	1	1	0	0	0	3	**63.6**	0.82±0.87	0	1	1	3	1	0	0	1	1	2	0	**63.6**	0.91±0.94
	Hemorrhage	0	0	1	1	0	0	1	0	0	0	0	27.3	0.27±0.47	0	0	0	2	2	0	0	1	1	2	0	45.5	0.73±0.9
	Edema	4	0	0	2	0	1	0	2	1	0	0	**45.5**	0.91±1.3	1	1	1	2	0	0	2	0	1	0	2	**63.6**	0.910.83
BLN	Fibrin	0	0	0	0	0	0	0	0	0	0	0	0	0	0	0	0	0	1	0	0	0	0	0	0	9.1	0.09±0.3
	Inflammation	1	0	0	1	0	0	1	0	0	0	0	27.3	0.27±0.47	0	0	0	0	0	0	2	1	2	1	1	45.5	0.64±0.81
	Hemorrhage	0	0	2	4	0	0	3	0	0	0	1	**36.4**	**0.91±1.45**	1	1	0	2	2	1	1	1	4	2	4	**90.9**	**1.73±1.3**
	Necrosis	2	1	2	3	2	2	3	1	0	2	2	**90.9**	**1.82±0.87**	2	2	1	2	4	3	2	2	4	2	4	**100**	**2.55±1**
	Edema	2	0	0	0	0	1	0	0	0	2	0	27.3	0.45±0.82	2	0	0	2	0	0	2	2	0	0	0	36.4	0.73±1
MnLN	Fibrin	0	0	0	0	0	0	0	0	0	0	0	0	0	0	0	0	0	0	2	0	1	0	0	0	18.2	0.27±0.65
	Inflammation	0	0	0	0	0	0	0	0	0	0	0	**0**	0	0	0	0	0	0	1	0	1	1	1	0	**36.4**	0.36±0.5
	Hemorrhage	0	0	0	0	1	0	0	0	0	0	0	9.1	0.09±0.3	0	0	0	0	0	1	0	0	0	1	1	27.3	0.27±0.47
	Necrosis	0	0	1	0	2	0	1	0	0	0	0	27.3	**0.36±0.67**	2	0	2	0	0	3	0	0	2	0	2	45.5	**1±1.2**
MsLN	Fibrin	0	0	0	0	0	0	0	0	0	0	0	0	0	0	0	0	1	0	0	0	0	1	0	0	18.2	0.18±0.4
	Inflammation	0	0	0	0	0	1	0	0	0	0	0	9.1	0.09±0.3	0	0	0	0	0	0	0	0	0	0	1	9.1	0.09±0.3
	Hemorrhage	0	0	0	0	1	0	0	0	0	0	0	9.1	0.09±0.3	0	0	0	0	0	0	0	0	0	0	0	0	0
	Necrosis	0	0	1	0	0	0	1	0	1	1	0	36.4	0.36±0.5	0	0	2	2	0	0	0	0	0	0	3	27.3	0.64±1.1
	Edema	0	0	0	0	0	0	0	0	0	0	0	0	0	0	0	0	0	0	0	0	1	0	0	0	9.1	0.09±0.3
Spleen	Fibrin	1	0	2	0	2	2	0	1	0	0	0	45.5	0.73±0.9	0	0	0	2	0	2	0	1	0	0	1	36.4	0.55±0.82
	Inflammation	2	2	0	2	0	0	1	1	0	1	3	**63.6**	**1.09±1.04**	0	1	2	0	2	1	2	3	1	3	2	**81.8**	**1.55±1**
	Hemorrhage	0	0	0	1	0	0	0	0	0	0	0	9.1	0.09±0.3	0	1	1	0	1	0	0	0	0	0	0	27.3	0.27±0.47
	Necrosis	1	1	2	2	2	2	2	2	2	0	2	**90.9**	**1.64±0.67**	1	0	2	2	0	0	0	2	0	2	1	**54.5**	**0.91±0.94**
Thymus	Edema	-	-	-	-	-	-	-	-	-	-	-	-	-	4	-	-	-	-	-	-	-	-	-	-	N/A	N/A
Gross Lesions	Codes											A			B					C	C			C, D	A,E,F		
	Incidence	0	0	0	0	0	0	0	0	0	0	1	**9.1**	0.1±0.3	1	0	0	0	0	1	1	0	0	2	3	**45.5**	0.7±1
	Total Pathology	15	5	14	19	12	12	16	7	6	10	14		**11.8±4.4**	16	8	13	24	15	24	21	19	23	33	31		**20.6±7.5**

Microscopic observations of bacteria (upper) and of anthrax-related pathologies (lower) were recorded for each animal. Organs evaluated microscopically in all animals listed by animal number (#), included brain, kidney, liver, lung, spleen, bronchial lymph nodes (BLN), mandibular lymph nodes (MnLN), and mesenteric lymph nodes (MsLN). Thymus was evaluated in animal 20 due to the presence of a gross lesion. Parameters scored semi-quantitatively included bacteria and other microscopic pathology, including fibrin, hemorrhage, inflammation, necrosis, and edema. Severity scores were minimal (grade 1, yellow), mild (grade 2, light orange), moderate (grade 3, dark orange) or marked (grade 4, red). A pathology score of 0 was included for microscopically normal tissues. The percent (%) of animals affected (incidence), and the mean severity scores with standard deviations (SD) were calculated by group for fast and slow progression. A score for total bacteria (Bact) and total pathology (Path) was calculated for each animal. Mean and SD for total bacteria and total pathology scores for fast and slow progression groups. The single surviving animal 23 did not have any pathologies consistent with anthrax and was excluded. **Bold** font indicates a high percentage (%) of animals affected and/or the highest one or two mean severity scores. **Bold underlined** fonts indicate parameters where the % affected or means were notably different between fast and slow progression. Cell type-specific necrosis involved lymphocytes in lymph nodes, lymphoid follicles in spleen, hepatocytes in liver, and renal tubules in kidney. Gross pathology findings (far right column) were coded as: A) 10-15 milliliters red fluid (ascites), B) 10 mL clear fluid in thymus, C) dark or red discoloration of meninges, brain, D) multiple dark liver foci, E) multiple pale liver foci, F) multiple enlarged, dark bronchial lymph nodes. Not applicable (N/A).

*Tissue bacteria and sepsis*. Animals in both groups had a high incidence (67–100%) of bacteria in organs and associated vascular spaces (Table 7). The spleen represented the greatest bacterial reservoir, with 21 animals affected and a severity grade of 3 or above in many animals. The two highest group mean severity scores for bacteria were in the spleen, averaging 3.1 (fast progression group) and 2.4 (slow progression group), followed by a mean severity score of 1.7 in the lungs in both groups. The only notable difference in bacterial tissue distribution between fast and slow progression animals was the presence of bacteria in brain in 81.8% of animals with fast progression and only 45.5% of animals with slow progression.

All but three of 23 animals presented with features of fatal sepsis, as defined by *B*. *anthracis* dissemination to non-lymphoid organs including kidneys, liver, and lungs. Of the three animals without apparent sepsis, one (animal 23, C58174) survived with no bacteria visible in any tissue. Two others, animals 18 (C58159) and 21 (C58156), in the slow progression group only had bacteria in the brain and lymphoid organs. Both animals had macroscopic observations of dark or red meninges that correlated with microscopic fibrin, inflammation, and hemorrhage. Additionally, seizures were observed in animal 18 prior to euthanasia, and terminal bacteremia in animal 18 and terminal toxemia in animal 21 were lower than those seen in animals with sepsis (Fig 3R and 3U). Together, these findings supported that death in these two animals was a consequence of meningitis rather than sepsis. A third animal in the slow progression group, animal 17 (C59617), also had macroscopic and microscopic evidence of meningitis but with concurrent sepsis, disseminated bacteria to all organs, and high terminal bacteremia and toxemia (Fig 3Q).

*Histopathology*. Among the tissues collected and processed for histopathological analysis, lung, bronchial lymph nodes, and spleen had the highest incidence of anthrax-related microscopic lesions, including edema, fibrin, inflammation, hemorrhage, and necrosis (Table 7). The incidence and severity of microscopic changes in the kidney and lung were comparable between fast and slow progression animals. Lesions in other organs often had higher incidence and/or severity among slow progression than fast progression animals. These included one or more of the following pathologies, fibrin, inflammation, hemorrhage, and necrosis, occurring in the brain, liver, bronchial, mandibular, and mesenteric lymph nodes, and spleen. The most notable of these with higher incidence and severity in slow progression was hemorrhage in the bronchial lymph nodes. Among fast progression animals, a higher incidence and mean severity of splenic lymphoid necrosis was noted as compared to slow progression animals.

Despite most fast progression animals having bacteria in the brain, tissue change was limited to one animal with minimal hemorrhage. There were more tissue changes in the brain of animals with slow progression, but they were limited to the three animals with meningitis. Meningitis was not seen in any animal that died less than 105 hours (4.4 days) post-challenge.

To facilitate global comparison between fast and slow progression animals, total bacteria scores were calculated for each animal by adding the individual severity scores for bacteria in all examined organs. Total pathology scores were calculated for each animal by adding the individual severity scores for all anthrax-related lesions (except for bacteria) in all examined organs including anthrax-related gross lesions. Group mean (±standard deviation) total bacteria scores were 11.7±2.5 for fast progression and 10.5±4.6 for slow progression. Conversely, group mean total pathology scores were 11.8±4.4 for fast progression and 20.6±7.5 for slow progression, demonstrating higher pathology overall for animals with longer survival times and thus, longer toxin exposure.

The single animal surviving to end-of-study (day 14) had non-specific pathology that included minimal chronic active inflammation in the lung and pale liver nodules, thought to be from a prior unrelated infection, with no microscopic evidence of anthrax.

## Discussion

*Anthrax progression*. Historically, clinical inhalation anthrax was described as a biphasic disease, with two stages of ‘illness’, early-prodromal and late-fulminant [3,48]. The first stage lasted several days and was followed by either direct progression to the late-fulminant stage or a period of improvement before entry to the late stage. During a 1957 outbreak, Plotkin *et al*. described five inhalation anthrax cases where after initial symptoms, one patient continued to work and two of the patients’ conditions were briefly ‘improved’ [48]. However, after onset of the final stage in four patients, they only survived 7 to 25 hours. The 2001 inhalation anthrax cases allowed refinement of the clinical description to include three stages: 1) the early-prodromal, 2) intermediate-progressive, and 3) the late-fulminant stage [7,33,34]. The kinetics of anthrax toxins determined previously in five rhesus macaques showed that PA, LF, LTx, EF, and the poly-γ-D-glutamic acid capsule kinetics were triphasic, with a rise, a plateau (or decline), and a final rise: potentially comparable to the three human clinical stages of anthrax [22,23,45,46]. This study allowed the first determination of all major anthrax toxins collectively through all stages of anthrax progression. The larger numbers of animals yielded variable progression compared to that seen in the five rhesus macaques.

Two primary patterns of toxemia emerged in this study revealing two distinct survival groups of similar sizes. In one group, animals had high early toxemia/bacteremia, faster disease progression, and early deaths. In the other group, animals had lower early toxemia, slower progression, and survived longer. Identifying the two survival groups allowed characterization and comparison of both types of infection progression in a way that has not been done before.

**Fig 7:**
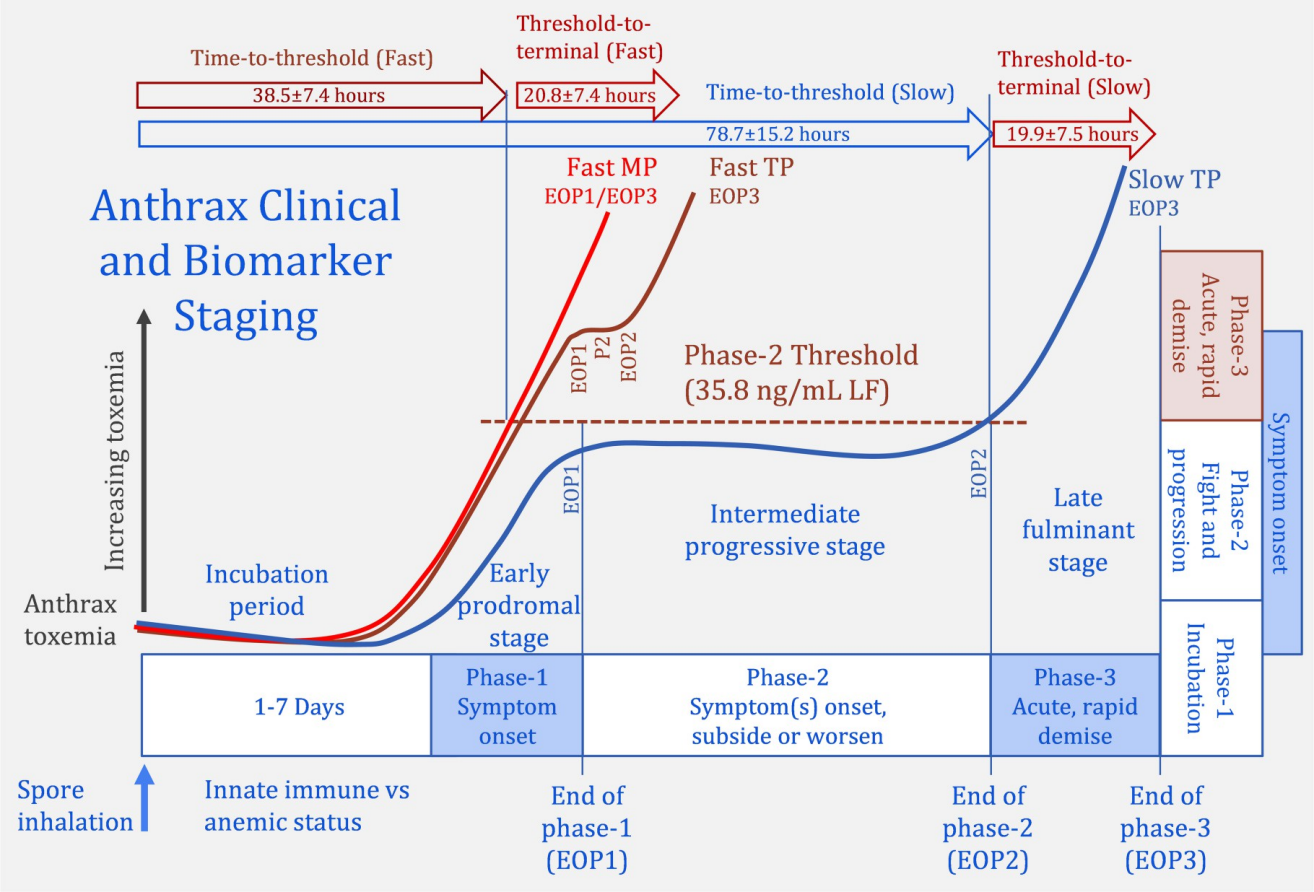
Proposed relationship of toxemia-defined progression with clinical staging. The triphasic (TP) kinetics of anthrax toxemia with slow progression in non-human primates mirrors the clinical staging algorithm. A brief incubation period is followed by 1) the early-prodromal stage 1 with the onset of non-specific symptoms which coincides with the early rise in toxemia to the end-of-phase-1 (EOP1). This is followed by 2) the intermediate-progressive stage which coincides with the plateau/decline in toxemia that continues to the end-of-phase-2 (EOP2) when the critical toxemic thresholds are exceeded, which coincides with entry to 3) the late-fulminant stage with a rapid decline in clinical stability and rapid rise in toxemia/bacteremia. Fast progression occurs over a compressed timeline during which the phase-2 plateau may (fast triphasic-TP) or may not be observed (fast monophasic-MP) kinetics. The dependence of progression on toxemia can be seen with the shorter time-to-threshold for fast progression than slow and the similar time from threshold-to-terminal for both. Progression may be reoriented from time/clinical-dependent staging to toxin/clinical-dependent staging.

Animals with slower progression exhibited a rise/plateau/rise, a triphasic kinetics, similar to that described in five rhesus macaques. The triphasic kinetics of slow progression can be aligned with the 3 clinical stages (Fig 7). Phase-1 begins with early toxemia, LF and EF, appearing during the incubation period, before symptom onset. LF and EF levels increase and other toxins, LTx, ETx, PA, and bacteremia appear, along with earliest symptoms. Toxins increase to the end-of-phase-1 (median levels of 23.8 ng/mL for LF). Phase-1 is analogous to the early-prodromal clinical stage-1. The end-of-phase-1 begins phase-2 where toxin levels plateau (PA/LF/LTx) or decline (EF/ETx/bacteremia) for an extended period, analogous to the intermediate-progressive stage-2. LF levels throughout phase-2 were relatively stable. The upper limits of this range defined a threshold above which phase-2 has ended and phase-3 commenced. After the plateau, toxins start to increase again. When toxin levels reached the threshold (35.8 ng/mL for LF), they entered phase-3, with rapid accumulation of toxemia and rapid demise, analogous to the late-fulminant stage-3.

For fast progression, the kinetics was either triphasic with a short phase-2 plateau, or monophasic, with the phase-2 plateau skipped, or not discernable within the early collection times. In monophasic progression the end-of-phase-1 and end-of-phase-3 are virtually synonymous. The kinetics of fast progression occurred within a compressed time frame (Fig 7). They reached higher levels at the end-of-phase-1 (138 ng/mL for LF), above the phase-2 threshold of 35.8 ng/mL for LF (and above thresholds for all toxins and bacteremia), indicating early entry to phase-3. Passing the critical thresholds early led to rapid death, even before symptoms in two animals.

After passing the phase-2 thresholds, toxin levels increased rapidly to the terminal stage. The mean time to reach the thresholds was twice as long in slow progression as fast, but the mean time from threshold to terminal was nearly identical for both groups at 20–21 hours. This time in phase-3 is consistent with the duration of stage 3 described in clinical cases [3,7,48,49]. The findings support reorientation of the progression scheme from a time-dependent to a toxin-dependent one (Fig 7). Evaluation of toxemia by survival time supports the critical toxin thresholds defined in this model, since no matter the time post-challenge, once the threshold is passed, terminal stage-3 follows rapidly.

The fast progression observed in almost half of the animals is a challenging problem since it leaves little time for successful treatment after symptoms. A rapid course can be suggested by evidence from the Sverdlovsk outbreak for which dates of both symptom onset and death were available for 50 cases [50]. The time from onset to death was on the same day for one case and within one day for 8 cases. A systematic review of inhalation anthrax also reported four cases with 1.5- to 1.6-days following initial symptoms [5]. Though the incubation periods and exposure doses are not certain, the rapid death following symptom onset suggest the possibility of rapid progression in humans.

*Other factors*. The weight-adjusted mean spore dose was higher in animals with fast progression than in those with slow progression, but the differences were not statically significant. Nonetheless, there was a higher average LD_50_/kg for animals with fast progression. Vasconcelos *et al*. also observed ‘a tendency’ for cynomolgus macaques that received higher spore doses to die more quickly than those that received lower doses [37]. This prompts consideration, if feasible, for tailoring animal study design to target the spore dose according to an animals’ body weight.

Early hematology showed trends related to time or progression. Time-dependent changes over the first 48 hours, increases in innate immune cells, such as white blood cells and neutrophils, and declines in red blood cells, hemoglobin, and hematocrit, occurred in both fast and slow progression, consistent with those seen in other animal models [51,52]. However, there were key differences between fast and slow progression. Means for innate immune markers, especially monocytes, were higher in slow progression, perhaps offering more defense in these early stages. Red cell distribution width (RDW) and corpuscular hemoglobin concentration mean (CHCM), both related to red cell fitness, were higher in animals with fast progression. High RDW is associated with deficiencies of iron, B-12, or folate and can be due to macrocytic anemia, underlying chronic inflammation, or infection [53]. Studies in middle aged and older adults showed that even modestly elevated RDW was associated with an increased risk of all-cause mortality [54]. Elevated RDW was also associated with increased severity of sepsis [55,56]. These factors may render the animals less resilient to the ensuing sepsis and advancing toxemia.

The anthrax toxins actions make the differences in these factors even more important. LTx is known to cause anemia and has also been shown to suppress erythropoiesis, exacerbating the anemia, and promoting progression [57]. Hemoglobin and hematocrit have been shown to drop during clinical inhalation anthrax as was observed here [58]. In this study, the lone survivor had higher white blood cells and lymphocytes, and exceptionally elevated red blood cell count, hemoglobin, and hematocrit. In contrast to animals with fast progression that had higher CHCM, the survivor also had the lowest CHCM (and MCHC) of all animals.

Two animals with long progression, animal 23 that survived and animal 22 that succumbed, had similar toxemic profiles, especially for LF, up to a point, but their toxemia eventually diverged with opposite outcomes. Differences in these animals that might have contributed to the opposite outcomes included the LD_50_ spore dose, which was higher in the survivor but when adjusted for weight was higher in the non-survivor. Despite that, toxin levels were similar, so you would expect to have similar toxic effects. Both had declines in RBC. However, the survivor started with a much higher RBC count. By 132 hours post-challenge, the RBC count was still within normal limits in the survivor but was below normal in the non-survivor. The higher beneficial hematological features described for the survivor may have provided a better level of defense against the organism and resilience against toxins for recovery.

*Pathological factors associated with disease progression*. Assessment of terminal-stage microscopic bacteria and pathology allowed evaluation of the burden and impact of anthrax resulting from the varying toxemic progressions. The general findings were consistent with those described for cynomolgus macaques previously, with primary involvement in the spleen, bronchial lymph nodes, and lungs [37]. While both groups had similar incidences of bacteria in the spleen, the severity of splenic bacteria was higher in animals with fast progression than slow progression. Both the incidence and severity of splenic lymphoid necrosis was almost twice as high in fast progression as in slow progression. Thus, both bacterial colonization and necrosis in the spleen were early to develop in rapidly progressing anthrax. Conversely, lesions in other organs often had higher incidence and/or severity among slow progression animals. The latter category included bronchial lymph node lymphocyte necrosis and hemorrhage, suggesting that longer toxin exposure may allow for lesion development.

Although the incidence of bacteria in the meninges was higher in fast progression than slow progression, the former had little to no associated brain pathology. Studies have indicated that ETx plays a role in disrupting the blood brain barrier (BBB) function and LTx in promoting microbial BBB penetration [59]. The higher early toxemia in fast progression may allow BBB penetration of bacilli, but death is rapid with little associated pathology. In animals with slow progression, the lower phase-2 toxemia may not facilitate penetration of the BBB as readily, but when bacteria did gain access, the longer toxin exposure may have contributed to more advanced pathology. These differences suggest the rapid sepsis of fast progression doesn’t allow sufficient time for a tissue response in the brain, whereas the longer course in slow progression allows for longer toxin exposure resulting in meningitis and death. Notably, meningitis was only seen in animals surviving at least 105 hours post-challenge. A following study in cynomolgus macaques included the analysis of toxin levels in terminal organ tissues to help understand the organ pathologies observed.

*Stage dependent toxemia*. The characterization of toxemia associated with the three stages of inhalation anthrax provides insights into the dynamics of these toxins throughout infection. In phase-1, the earliest stages of infection before symptom onset, detection was limited to LF and EF. LF was the best biomarker for early, pre-symptom onset detection based on its early detection and abundance. LF was previously measured in human cutaneous anthrax in the absence of sepsis [60]. In these cases, it appears that LF is secreted locally from the site of infection, the cutaneous lesion, and detected in circulation. Early detection of LF during inhalation anthrax, before bacteria are detectable in the blood, may originate from early events, such as germinating spores within macrophages [61].

As the earliest secreted toxins start to act, LF and EF levels rise to the end-of-phase-1 when other toxins and bacteremia become detectable. The analytic sensitivity of the catalytic toxin methods allowed LTx and ETx to be detected before total-PA and concurrent with bacteremia. Both LTx and ETx comprise a fraction of the available PA63 and signal the point when active toxin complexes begin to accumulate. The mean first detection of LTx at 30.5 hours coincides with that of bacteremia at 30.5 hours which included low ‘+’ bacteremia below the LOQ. The first detection of ETx at 33.4 hours closely coincides with the first detection of quantifiable bacteremia at 34.2 hours. This suggests that toxin complexes (LTx and ETx) are detectable in blood at the same time as detectable and measurable bacilli, respectively.

PA, the most abundant toxin once positive, was detected latest, and had more non-detectable results at symptom onset than other biomarkers. The later detection of PA may be due in part to the higher detection limit and potentially, the rate of cellular uptake of PA during early stages, resulting in less PA in circulation. PA83 was only detected sporadically and was most consistently positive at the highest levels of total PA. This indicates that PA83 is rapidly hydrolyzed and activated to PA63, throughout infection, as was seen in rhesus macaques [45]. The scarcity of PA83 is most likely due to the activity of serum proteases that rapidly cleave PA83 to PA63 which then binds free LF and EF, forming active toxin complexes [19]. Some PA83 may also be removed by binding to cell surface receptors, where it is proteolytically activated to form oligomers that bind LF and EF [62]. PA83 is only detectable when its production is in excess relative to these processes. As such, any tests for PA83 alone are not useful for diagnosis or for predicting anthrax stages.

Phase-2 of slow progression was the most important feature characterized here, since once it is passed, death followed rapidly, irrespective of time. Phase-2 corresponds with the intermediate-progressive clinical stage where the host battles the organism and the effects of intoxication begin to accumulate but have not yet surpassed host defenses. Of all the toxins, LF was the least variable during this period. PA and LTx, were also less variable. The limited range of concentrations of LF during phase-2 suggest the possibility that levels are somewhat controlled, either by the host or organism (or both), maintained below the threshold until host defenses are overcome. Bacteremia, EF, and ETx were the most variable during phase 2. Lower levels seen for EF and ETx may be influenced more by the fluctuations in bacteria and dynamics of cellular intoxication during phase-2.

An early study by Smith and Keppie identified a bacteremia threshold in the guinea pig model, a point of no return, above which antibiotic treatment failed [11]. In clinical inhalation anthrax, treatment commencing in the late-fulminant stage 3 had the lowest cure rate, 97% fatality for a century of cases [5]. Thus, clinical stage-3, with the highest treatment failures, is essentially analogous to conditions beyond the bacteremia threshold in the guinea pig model, when treatment fails. Clinical stage-3 is also analogous to phase-3 in this cynomolgus macaque model, indicating the phase-2 thresholds for entry to phase-3 may predict stage-3-dependent treatment failures.

The threshold for bacteremia defined here can be compared to the critical level for antimicrobial treatment failures determined previously [11,63]. Using intradermal *B*. *anthracis* infection in the guinea pig model, showed that animals recovered if treated when bacteremia was 0.7 to 1.5x10^6^ cfu/mL and died when it was greater than 3x10^6^ cfu/mL. The threshold for bacteremia in cynomolgus macaques at 1.5E+05 cfu/mL was 20-fold lower than that described in guinea pigs. This is not surprising for different models with different infection routes. We have similar toxin results in other animal models, rhesus macaques and New Zealand White rabbits. They both show a triphasic kinetics from which phase-2 thresholds can be estimated. Comparing the differences between animal models might reveal how the thresholds can be applied to human inhalation anthrax.

In phase-3, whether fast or slow progression, toxins and bacteremia increase rapidly. Toxin levels and bacteremia higher than the thresholds suggest there is less than 20 hours of life remaining. The higher the levels, the shorter the interval to death. Smith and Keppie described an inverse relationship between the bacterial count in the blood and the time-dependent interval to death [11]. We observed a similar relationship in cynomolgus macaques, a negative correlation of LF with survival time. We plan to explore this correlation further. We will also test whether the phase-2 thresholds, indicating an animal is in phase-3, represent the antimicrobial point of no return where treatment fails.

The stages of anthrax can be deduced from toxin levels and toxin ratios. In phase-1, lower toxin levels correlate with higher toxin ratios (divergent toxemia). Conversely, in phase-3 higher toxin levels correlate with lower toxin ratios (convergent toxemia), which may be reached early or late. All toxin ratios fit this pattern except for PA/LF. The threshold for LF/LTx was 2.5–5.2 in early stages, declined to 1.7 at the phase-2 threshold, then declined further to 1.2 at terminal. It was even as low as 1.0 (100% LTx) in animals with the highest toxemia, indicating that most of the LF is present as LTx at terminal stage. Except for the rare instances of advanced meningitis with lower terminal toxemia, high toxemia with low toxin ratios was the most consistent indicator of terminal stage inhalation anthrax with sepsis. Collectively, the findings in this study detailing toxemia, bacteremia, hematology, and pathology, for diverse disease progressions, may allow strategies for targeted therapeutics and a model for advanced interpretation of outcomes.

## Conclusions

This assessment of all major anthrax toxins and bacteremia, along with hematology and pathology allowed a comprehensive evaluation of inhalation anthrax in the cynomolgus macaque NHP model. We defined a triphasic kinetics of toxemia which was aligned with the three clinical stages of anthrax. This indicated that anthrax progression is toxin-dependent rather than time-dependent. Hematological factors may influence the disease course. Lethal factor represented the most significant indicator of anthrax infection and progression with a remarkably invariable phase-2, such that a small increase potentially advances infection to phase-3 and rapid demise. Phase-2 thresholds represent a potential point of no return which might predict antimicrobial treatment failures. Comprehensive toxin analysis in studies with combined antimicrobial and antitoxin treatment is essential to advance our understanding of anthrax in humans and when specialized treatments are most efficacious.

## Methods

### Ethics statement

The animal study protocol was approved (approved protocol number 3013), by the Battelle Memorial Institute’s Institutional Animal Care and Use Committee (IACUC), Battelle PHS Approved Animal Welfare Assurance ID: D16-00021 (A3034-01), following Health and Human Services (HHS), National Institutes of Health (NIH), Office of Laboratory Animal Welfare principles.

### Reagents

Materials and reagents were obtained from Sigma-Aldrich (St Charles, MO) except where indicated. LF, PA83, and PA63 were obtained from List Biological Laboratories (Campbell, CA), and EF was from Dr. Stephen Leppla’s laboratory (National Institutes of Health, National Institute of Allergy and Infectious Diseases) as described [23]. All other reagents, materials and methods for anthrax toxin measurements were described previously [22,23,44,45,64].

### Safety and quality

The animal exposure study was conducted in 2014 at Battelle Biomedical Research Center (Columbus, OH) in a BSL3 secured laboratory registered with the Centers for Disease Control and Prevention (CDC) for select agents and inspected by the Department of Defense and U.S. Department of Agriculture. All requirements for select agent laboratories and handling of *B*. *anthracis* were followed. Battelle performed all aspects of the animal study as well as sample analyses for quantitative bacteremia and hematology as described in detail below. Samples sent from Battelle to the CDC for analysis of anthrax toxins were filter sterilized and sterility confirmed prior to shipment. Sample analysis at CDC was performed in a BSL2 laboratory and followed all safety requirements for handling biological specimens. All personnel have full training required for safety and follow all general laboratory, chemical and bloodborne pathogens procedures. Chemical hygiene plans are in place. All sample analyses followed high quality procedures using validated methods and conducted in a CLIA-certified laboratory following strict adherence of CLIA regulations. Battelle follows good laboratory practice guidelines as available in their standard operating procedures and facility practices.

### Animal study

The animal exposure study was conducted at Battelle (Columbus, OH), a Public Health Service (PHS) Animal Welfare Assurance approved facility (OLAW Assurance ID: D16-00021). The study protocol was approved by the Battelle Institutional Animal Care and Use Committee (IACUC) (Approved Protocol #3013) as required by PHS policy following Health and Human Services (HHS), National Institutes of Health (NIH), Office of Laboratory Animal Welfare principles. Work with animals followed all safety and select agent guidelines described above. All aspects of the animal study protocols were designed to minimize stress in the animals. CDC received study samples from Battelle for toxin analysis and was not involved with any component of the animal protocol. Chief of CDC Animal Care confirmed that the use that Battelle IACUC was sufficient.

Twenty-three *Macaca fascicularis* cynomolgus macaques (12 males, 11 females) from Covance, Inc., Alice, TX, weighing 3.1–4.9 kg prior to spore exposure, were confirmed to be healthy, negative for tuberculosis, seronegative for Simian Immunodeficiency Virus, Simian T-Lymphotropic Virus-1, and Macacine herpesvirus 1 and PCR negative for Simian Retrovirus 1 & 2. Animals were quarantined for 5 weeks in paired housing, then acclimated to single housing in the biosafety level 3 (BSL3) laboratory one week before exposure.

This study was conducted in two challenge groups with the first exposures (Group-1) taking place in November 2013 and the second in February 2014 (Group-2). Group-1 included 10 animals of which 8 (4 males, 4 females) were challenged and 2 (1 male, 1 female) were not challenged (naïve controls). In Group-2, 15 CM (8 males, 7 females), which included the 2 controls from Group-1, were challenged. Spore challenge exposures in this model were performed as described previously with a target dose of 200 median lethal dose (LD_50_) *B*. *anthracis* Ames spores via head-only inhalation exposure, and measurement of actual spore dose per animal by plethysmography [37,42].

Animals were routinely monitored, and clinical signs recorded twice-daily pre-challenge and every 6±1 hours commencing at 12 hours post-challenge. The following clinical signs of anthrax were monitored, anorexia, lethargy, respiratory distress, moribundity, abnormal activity such as recumbency, weak, or unresponsive, as well as seizures or any other abnormal signs. Signs of symptoms recorded included normal (N), hunched posture (HP), no stool (NS), soft stool (SS), diarrhea (DI), vomiting (V), lethargic (L), seizures (SE), labored breathing (LB), wheezing (W), moribund (M), unresponsive (UR), prostrate (P). Animals found to be moribund were humanely euthanized. Time to symptom onset was the difference in time hours and minutes from challenge to the hours and minutes of first specific clinical sign described from the 6-hour monitoring during the post-challenge period. NS was not included as a specific sign since it was also described prior to exposure and was not specific.

Body temperatures for all animals including group-1 controls were monitored via an implantable programmable temperature transponder (IPTT-300 BMDS, Seaford, DE), one each between the shoulder blades and left hip. Temperatures were recorded daily from day -7 through 0 (before exposure) and every 6±1 hours post-challenge. The threshold for a significant increase in body temperature (SIBT) was previously established as the mean pre-challenge (or baseline) temperature plus 2 standard deviations (SD) of the pre-challenge temperature [47].

### Sample collection

Samples collected for Group-1 included whole blood for quantitative bacteremia and plasma for toxins in EDTA tubes at -7 days pre-exposure (-7D) and post-exposure at 18-, 24-, 30-, 36-, 48-, 60-, 72-, 84-, and 96-hours and terminal sample when possible. Plasma sample collection was adjusted for Group-2 animals to include a 42-hour collection time and additional samples at 108-, 120-, 132-, and 144-hours and end of study (day 14). Whole blood for critical blood counts (CBC) (hematology) was collected in EDTA tubes for Group-1 animals at -7D, 18-, 24-, 30-, 36-, 48-, and 72-hours and for Group-2 at -7D, 18-, 24-, 30-, 36-, 48-, 72-, 84-, 96-, 108-, 120-, and 144-hours post-challenge.

### Quantitative bacteremia

The concentration in colony forming units (CFU) of *B*. *anthracis* vegetative bacteria per mL of whole blood was determined by serial dilution, plating and colony enumeration as described previously [65]. Briefly, 100 μL each whole blood sample was diluted in 900 μL phosphate-buffered saline (PBS), then serially diluted 10-fold in PBS up to 10^7^. Then 100 μL of each sample and dilutions were plated in triplicate onto Tryptic Soy Agar, incubated at 37°C for 16–24 h. Mean colony counts from 25–250 cfu/mL, below the quantitative range, were approved for reporting by the Study Director and used here as reported. The quantitative LOD was 100 cfu/mL. Values down to 25 cfu/mL when reported were included. A result of ‘+’ indicated that the mean colony counts for a dilution set (2 of 3 plates) was less than 10. For within group quantification, such as at symptom onset, values of ‘+’ were assigned the qualitative bacteremia LOD of 6 cfu/mL [46] and negative samples were assigned a value at ½ the LOD, 3 cfu/mL.

### Hematology

Hematology, CBC (whole blood) measurements were determined at Battelle with the Advia 120 (Siemens, Deerfield, IL). Measurements included, white blood cell count and differential leukocyte count (absolute and percentage), hemoglobin, hematocrit, red blood cell count, mean corpuscular volume (MCV), mean corpuscular hemoglobin, mean corpuscular hemoglobin concentration (MCHC), corpuscular hemoglobin concentration mean (CHCM), red cell distribution width (RDW), platelet count, mean platelet volume (MPV), neutrophil:lymphocyte ratio (N/L ratio).

### Mass spectrometry (MS) methods for anthrax toxins

MS methods for anthrax toxins were conducted at CDC. Validated methods for analysis of total-LF and LTx have been described previously [22,44]. Activity of EF monomer and ETx (PA_63_-EF) are congruent and were analyzed for EF activity as described [23]. Analysis of total-PA and PA83 for select tryptic peptides in the PA20 and PA63 domains was also performed as described [45]. The limits of detection for all the anthrax toxin methods are included in Table 2. Performance characteristics are detailed in the method publications [22,23,44,45].

Anthrax toxin MS methods provide high sensitivity, specificity, and accurate measures of all anthrax toxins [22,45]. Magnetic monoclonal antibody bead capture allows selective immunoprecipitation of toxins, (Step 1), followed by endoproteinase activity of LF/LTx, adenylate cyclase activity of EF/ETx and on-bead tryptic digest for PA (Step 2), and detection of LF/LTx and EF/ETx catalytic products and PA tryptic peptides by isotope-dilution MS (Step 3). The detection of catalytic products of LF, LTx, EF and ETx activity, facilitates signal amplification and enables exquisite analytic sensitivity. LC-MS/MS quantification of specific PA tryptic peptides allows accurate quantification of both total-PA and PA83, and determination of the amount of active PA63. These methods were used to measure toxins in cynomolgus macaques throughout infection. Throughout this report, total LF is referred to as LF and the amount of LF in complex with PA63 as LTx. Similarly, total EF is EF and in complex as ETx. Total PA is described as PA and the individual components as PA83 or PA63.3

### Pathology

All cynomolgus macaques, including the survivor to the end of the study, underwent complete necropsy with recording of gross observations. The brain, kidney liver, lung, bronchial, mandibular, and mesenteric lymph nodes, spleen, and gross lesions were collected and preserved in 10% neutral buffered formalin. Preserved tissues were trimmed, paraffin-embedded, sectioned to approximately 5 microns in thickness, affixed to glass slides, and stained with hematoxylin and eosin for brightfield microscopic examination by a board-certified veterinary pathologist. Microscopic findings were graded semi-quantitatively using the following scale: minimal (grade 1) for a minimally detectable lesion, mild (grade 2) for an easily visualized lesion, moderate (grade 3) for a lesion affecting larger areas of the tissue and marked (grade 4) for lesions affecting maximum areas of the tissue. Group summary incidence and average severity calculations were performed for fast and slow progression cohorts. Total Bacteria scores were calculated for each animal by adding the individual microscopic severity scores for bacteria in all examined organs. Total Pathology scores were calculated for each animal by adding the individual microscopic severity scores for all anthrax-related lesions (except for bacteria) in all examined organs as well as the incidence of any gross lesions in the animal.

### Statistical analysis and reporting

Toxin levels for total LF (LF), LTx, total EF (EF) and ETx were measured and reported in ng/mL. Total PA (PA) and PA83 were measured in pmol/mL and converted to ng/mL. Calculations of total PA, PA63 and PA83 were determined as follows: In pmol/mL total PA minus PA83 gives PA63 (pmol/mL). PA83 (83 kDa) and PA63 (63 kDa) pmol/mL value multiplied by unit adjusted 83 and 63 ng/pmol, respectively give ng/mL values each which were added to give total PA (PA63+PA83) in ng/mL. Total PA is referred to as PA and any references to PA63 and PA83 are specified.

All calculations of mean, standard deviation, median and interquartile range (IQR), 95 percent confidence intervals, and other analyses where reported were performed using standard equations in Microsoft Excel or JMP, Version 15, SAS Institute Inc., Cary, NC, 1989–2019. Levels of toxins and bacteremia at specific time points or stages often varied by multiple logs and were skewed (nonparametric). Median and interquartile ranges accurately represented the center of data and range. Mann Whitney Wilcoxon Rank Sum Test (Wilcoxon/Kruskal-Wallis Rank Sum Test in JMP) was used to test the probability that two populations are equal for toxin levels between animals with fast vs slow progression [66]. P-value (Prob>|Z|) is reported for the normal approximation test for the two groups (Z test statistic. Alpha level set at 0.05.

For hematological parameters, time post-challenge was limited to 48h because only two animals with fast progression had later measurements, prohibiting comparisons beyond 48h. This demonstrates early changes and differences that may contribute to fast and slow progression. Oneway ANOVA was used to compare differences in means between animals with fast and slow progression at specific time points. JMP Fit model was used to test the effect of hematology over the time period day -7 pre-challenge, 24-, 36- and 48-hours and for progression (fast vs slow). P-value reported for each term was prob > |t| (t-ratio) and for overall effect tests was prob > F (F-ratio). Alpha level set at 0.05

### Progression determination and kinetics of toxemia

Animals fell into two groups defined by the highest toxin levels and bacteremia reached by 48 hours. Toxemia/bacteremia was higher in 11 animals that died early (fast progression) and were lower in 11 animals that survived longer (slow progression) (n = 11) and one animal that survived to the end of the study. In four of 23 animals the initial phase-1 rise proceeded directly to terminal phase-3 (monophasic). The phases were primarily triphasic, consisting of an initial rise (phase-1), plateau/decline (phase-2), and final rise (phase-3). Of all biomarkers measured, LF appeared earliest and was the most consistent (least variable) biomarker during the phase-2 plateau. Therefore, the kinetics of LF was used as the model to estimate the stages of each animal. This estimation is limited by the collection time points. Bacteremia was used to evaluate the kinetics for missing toxin measurements at terminal time points.

Phase-1 was defined by the time of first detection of LF during which it rises to a first peak, the end-of-phase-1, before the plateau or decline. The end-of phase-1 was defined as the time point when biomarker rise slowed or stopped and consequently begins phase-2. In phase-2 the phase-2 plateau continued until the final rapid rise to terminal (phase-3). Phase-3 represents the end of the disease course at (or near) death/euthanasia. Except for bacteremia, terminal toxin levels can only be estimated ‘near’ terminal since in each animal, final samples depended on the symptoms and time of euthanasia. Also, unpredictability and speed of final progression sometimes meant end-stage plasma samples were not available. A terminal whole blood sample for bacteremia was collected even when a plasma sample was not.

Note that some animals were missing a sample collection related to a specific stage. Four animals had no phase-2 (monophasic), of which one animal had no sample for toxemia after 36h (no terminal). Two animals with long survival had a long collection gap between the sample prior to the terminal time and terminal samples. Many were missing terminal plasma for toxins. For phase-3 terminal calculations, only a sample measured ≤12h from the death/euthanasia time point were included.

### Phase-2 and phase-2 threshold

In slow progression, the low variability of LF over the duration of phase-2 (< 1-order of magnitude) allowed characterization of this stage as a whole., Phase-2 was characterized for all toxins, bacteremia, and toxin ratios and included all measurements for all time points from the end-of- phase-1 to the end-of- phase-2 from all 11 animals (n = 52, 51 for ETx). The median, upper and lower quartiles, and 95% interval (upper limit) for toxins/bacteremia and 5% interval (lower limit) for toxin ratios were determined in JMP. The phase-2 upper 95% intervals represent potential critical toxin thresholds, which when exceeded signal departure from phase-2 and entry to phase-3. Lower 95% interval applies to toxin ratios.

### Time to threshold and time from threshold to terminal

The time to threshold was calculated from a linear regression for the two time points between which the threshold fell for all animals with available points. Animals with phase-2 levels within the typical standard error (≤10%) of the threshold for LF and followed with lower levels for an extended phase-2, the threshold was determined not to have been exceeded and was calculated at the later time when the final rise continued. Five animals were excluded from the threshold calculations. For one animal with fast progression and one with slow progression the threshold was not exceeded, but later (terminal) samples were missing so the time to threshold could only be calculated for bacteremia (Fig 3A and 3S). Animals 21 and 22 with slow progression had large time-gaps in sample collection (44.1 and 58 hours) between the last sub-threshold level sample and final/terminal samples, such that calculation of the time to threshold could not be estimated. The survivor (animal 23) never exceeded the threshold. Two animals (Fig 3G and 3K) were excluded from calculations for PA and ETx because they were below the PA, ETx detection limit before the sample that exceeded the threshold. An n = 8 is indicated for the fast group for PA and ETx, versus n = 10 for other toxins. Mean time to threshold was calculated for 8–11 animals with fast progression and 8–9 animals with slow progression (exact numbers-n in Fig 5). The time from threshold to terminal was determined by subtracting the calculated time to threshold from the terminal time, estimating how long an animal lived after reaching the threshold (time in phase-3).

## Supporting information

S1 Data File Detailed data with associated information used to generate table and graphs (where not already provided in the main article). (XLSX)

S2 Data File All anthrax toxin results in cynomolgus macaques with inhalation anthrax. (XLSX)

S3 Data File All bacteremia results in cynomolgus macaques with inhalation anthrax. (XLSX)

S4 Data File All hematology results in cynomolgus macaques with inhalation anthrax. (XLSX)

S1 Fig Survival curve for 23 cynomolgus macaques with inhalation anthrax. Proportion surviving vs survival time (hours).
(TIF)

S2 Fig Significant increase in body temperature post-challenge. The threshold for a significant increase in body temperature (SIBT) is established as the mean pre-challenge (or baseline) temperature plus 2 standard deviations (SD) of the mean pre-challenge temperature. Left hip and right shoulder transponders recorded temperatures in °F once daily for seven days. The pre-challenge mean, SD, and threshold (mean+2 SD) was determined for both the shoulder and hip transponders for each animal. The deviation from the pre-challenge threshold was calculated by subtracting the normal threshold temperature from the post-challenge temperatures. The solid horizontal line represents the thresholds with zero for the threshold. Deviations from threshold for shoulder and hip graphed for each animal by number according to survival time. Group-1 controls indicated with -1 (C58003) control corresponding to group 2 challenged animal number 5 and 0 (C58170) corresponding to group 2 challenged animal number 11.
(TIF)

S3 Fig Kinetics of anthrax biomarkers in cynomolgus macaques with slow progression. All toxins (ng/mL) and bacteremia (cfu/mL) over the course of infection in 12 animals with inhalation anthrax and slow progression, of which 11 were triphasic with phase-2 ≥24 hours and one with phase-3 decline and survival. One negative value at the end-of-study (EOS) (animal 23 -C58174) was graphed at ½ the limit of detection for each method as indicated in Table 2. For bacteremia (bact), non-quantifiable results of + was assigned a 6 (+ symbols) and negative at 3 cfu/mL (x symbols). The last time point for C59618 at terminal (Term, 8.4 days) and the survivor which declined to less than the limit of detection at the end-of-study (EOS, 14-days/336 hours) are not to scale. Black boxes represent the range of levels during the phase-2 for each biomarker.
(TIF)

S4 Fig Relationship between anthrax biomarker levels and survival time (days). Mean log_10_ concentrations and standard deviations (error bars) for total LF, LTx, total EF, ETx, total PA, and bacteremia, over the time post-challenge (hours-h) for animals that died/euthanized at 1.9–3.0 days (n = 10), 3.1 to 4.0 days (n = 5), 4.1 to 5 days (n = 4) and 5.1 to 6 days (n = 2), and for one animal at 8.4 days (animal 22) and survivor to end-of-study (End) (animal 23). Final time points for animal 22 (C59618) at 8.4 days and for 23 (C58174) at end-of-study (14 days) were included together (End). Gray dashed lines shown for median phase-2 level and phase-2 thresholds (red dotted line).
(TIF)

S1 Table End-of-phase-1 toxins, bacteremia, and toxin ratios. Individual results at the end-of-phase-1(P1) are given for animals with high toxemia, low ratios, and early time-to-death (fast progression) and animals with low toxemia, high ratios, and later time-to-death. Median, upper quartile, and lower quartile, determined in JMP. P-values comparing fast and slow progression obtained by Mann-Whitney-Wilcoxon rank sum (non-parametric) test. Levels for survivor shown separately. *Still in phase-1, excluded. **Less than the limit of detection (<LOD) for PA83 (1.22 ng/mL) given a value at ½ times the LOD for PA83 (0.61 ng/mL) for calculations.
(PDF)
