# Supplementary figures and images for: Comprehensive characterization of toxins during progression of inhalation anthrax in a non-human primate model
Source: PLoS Pathog. 2022 Dec 19;18(12):e1010735. doi: 10.1371/journal.ppat.1010735 (PMC9810172; doi:10.1371/journal.ppat.1010735)

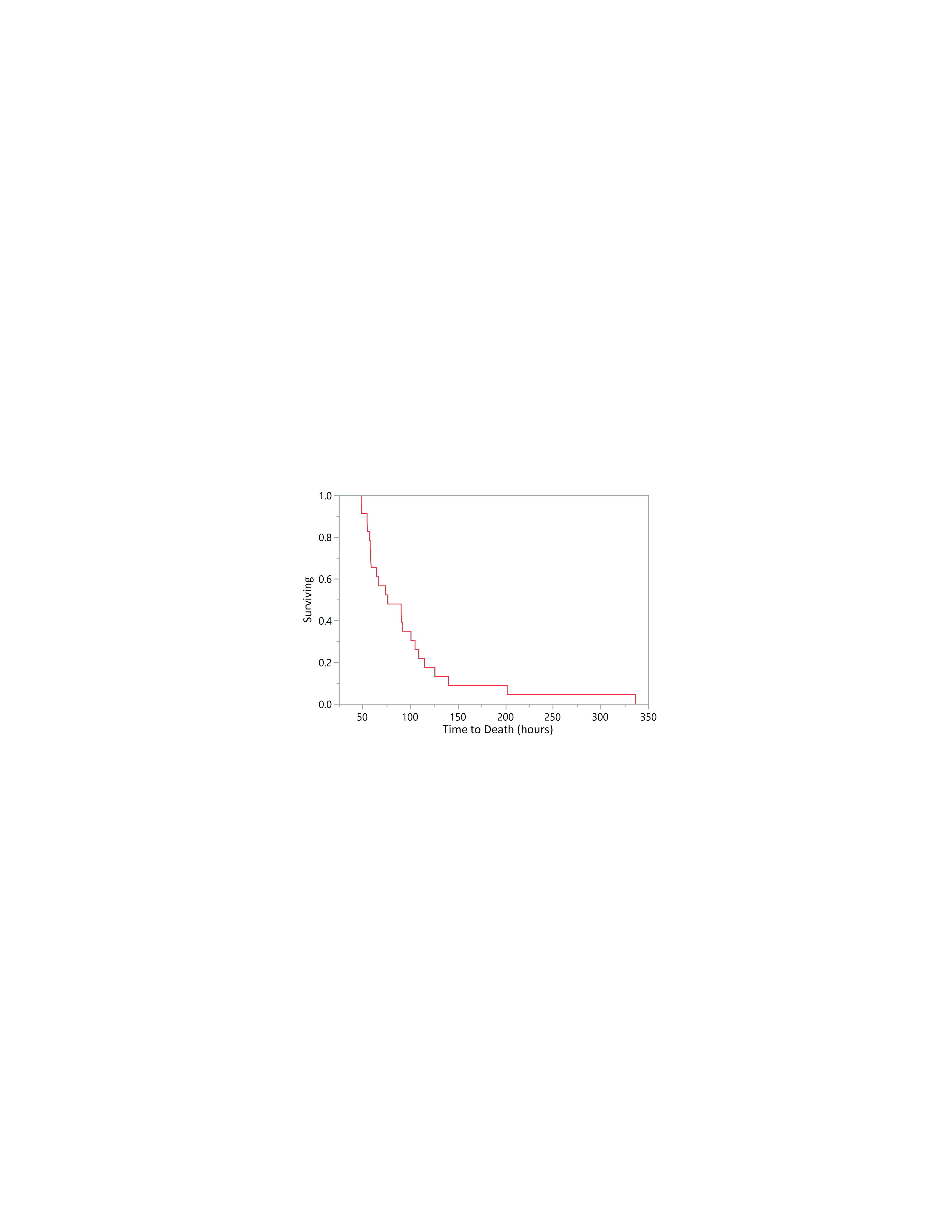

Supplement: S1 Fig — Proportion surviving vs survival time (hours). (TIF) [file ppat.1010735.s005.tif]

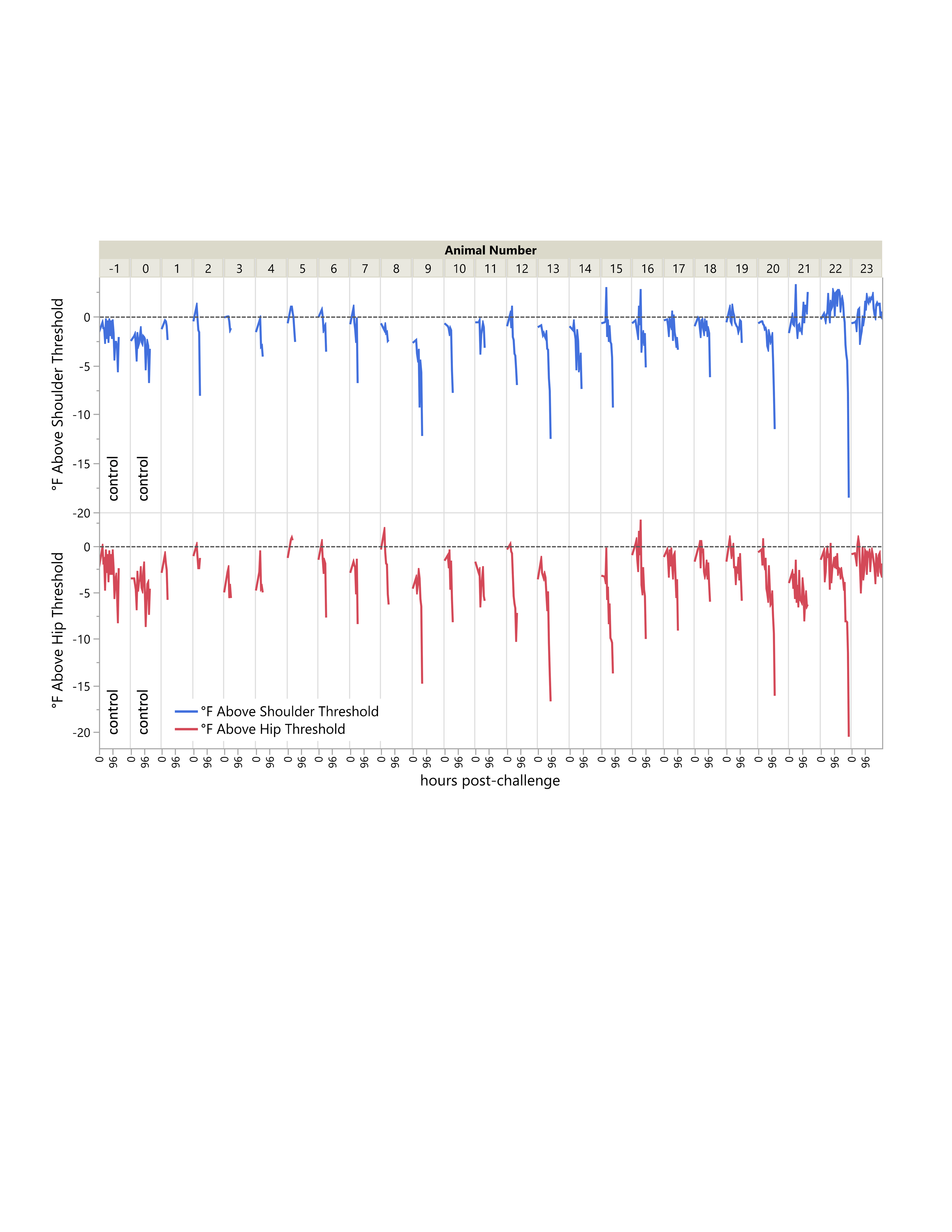

Supplement: S2 Fig — The threshold for a significant increase in body temperature (SIBT) is established as the mean pre-challenge (or baseline) temperature plus 2 standard deviations (SD) of the mean pre-challenge temperature. Left hip and right shoulder transponders recorded temperatures in °F once daily for seven days. The pre-challenge mean, SD, and threshold (mean+2 SD) was determined for both the shoulder and hip transponders for each animal. The deviation from the pre-challenge threshold was calculated by subtracting the normal threshold temperature from the post-challenge temperatures. The solid horizontal line represents the thresholds with zero for the threshold. Deviations from threshold for shoulder and hip graphed for each animal by number according to survival time. Group-1 controls indicated with -1 (C58003) control corresponding to group 2 challenged animal number 5 and 0 (C58170) corresponding to group 2 challenged animal number 11. (TIF) [file ppat.1010735.s006.tif]

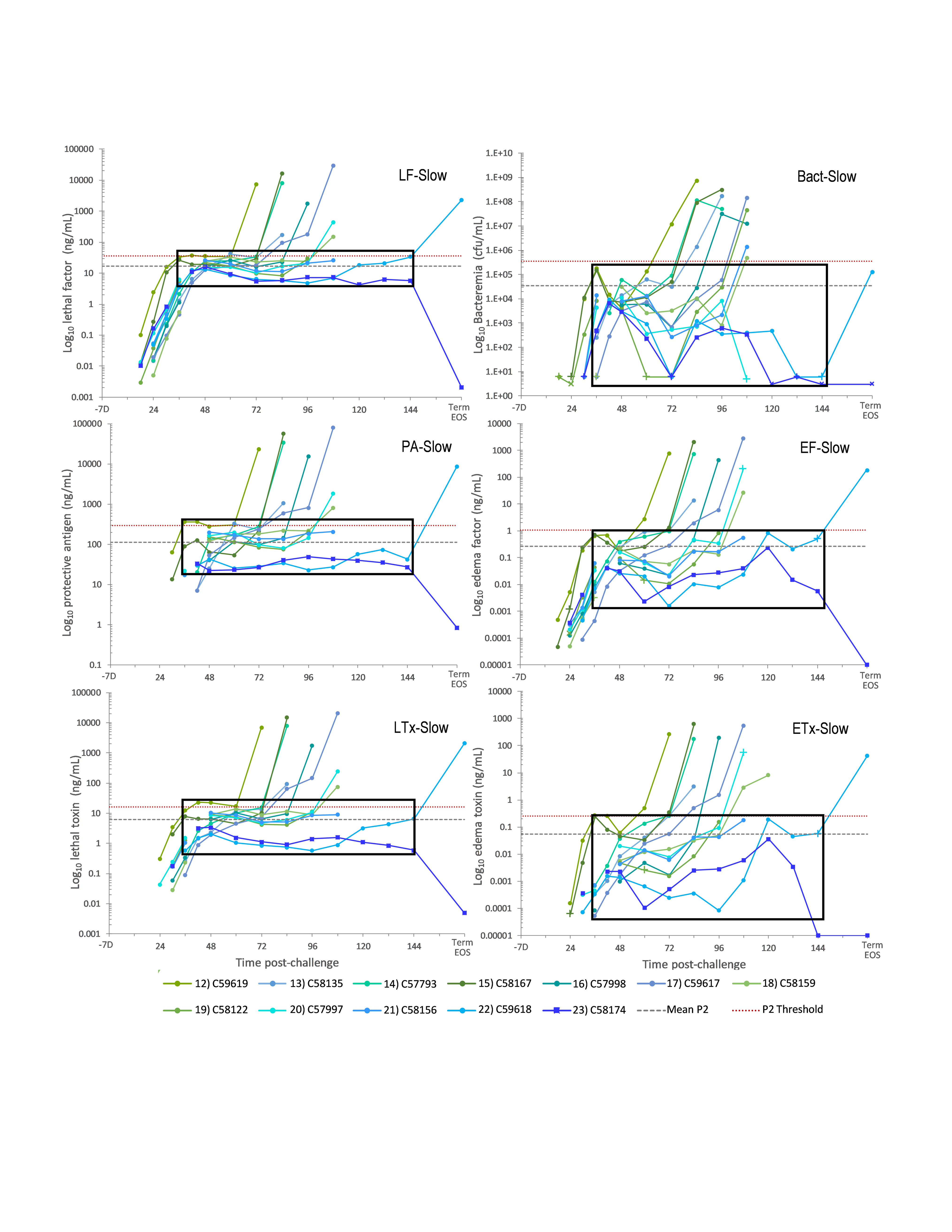

Supplement: S3 Fig — All toxins (ng/mL) and bacteremia (cfu/mL) over the course of infection in 12 animals with inhalation anthrax and slow progression, of which 11 were triphasic with phase-2 ≥24 hours and one with phase-3 decline and survival. One negative value at the end-of-study (EOS) (animal 23 -C58174) was graphed at ½ the limit of detection for each method as indicated in Table 2. For bacteremia (bact), non-quantifiable results of + was assigned a 6 (+ symbols) and negative at 3 cfu/mL (x symbols). The last time point for C59618 at terminal (Term, 8.4 days) and the survivor which declined to less than the limit of detection at the end-of-study (EOS, 14-days/336 hours) are not to scale. Black boxes represent the range of levels during the phase-2 for each biomarker. (TIF) [file ppat.1010735.s007.tif]

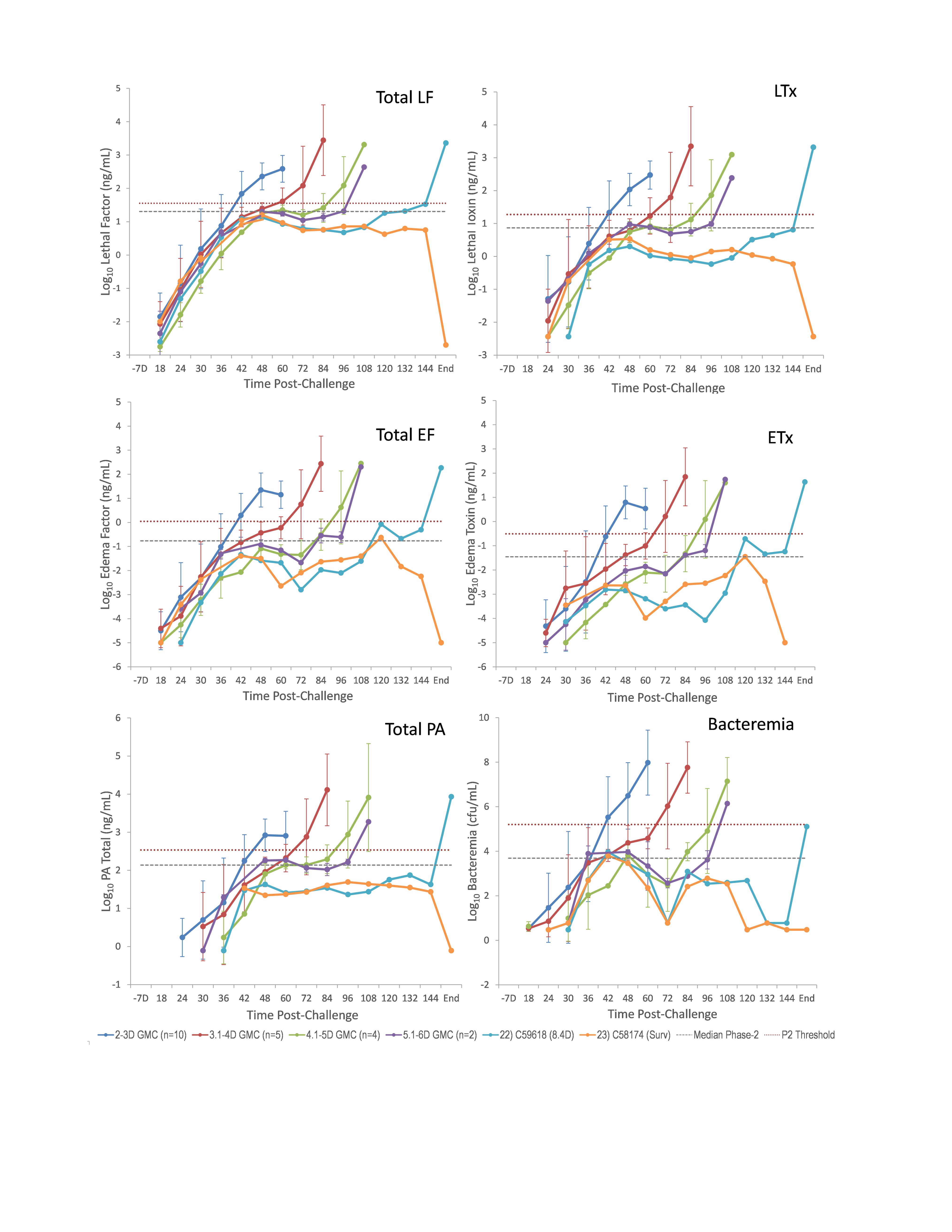

Supplement: S4 Fig — Mean log10 concentrations and standard deviations (error bars) for total LF, LTx, total EF, ETx, total PA, and bacteremia, over the time post-challenge (hours-h) for animals that died/euthanized at 1.9–3.0 days (n = 10), 3.1 to 4.0 days (n = 5), 4.1 to 5 days (n = 4) and 5.1 to 6 days (n = 2), and for one animal at 8.4 days (animal 22) and survivor to end-of-study (End) (animal 23). Final time points for animal 22 (C59618) at 8.4 days and for 23 (C58174) at end-of-study (14 days) were included together (End). Gray dashed lines shown for median phase-2 level and phase-2 thresholds (red dotted line). (TIF) [file ppat.1010735.s008.tif]
