# Supplementary material for: Comprehensive characterization of toxins during progression of inhalation anthrax in a non-human primate model
Source: PLoS Pathog. 2022 Dec 19;18(12):e1010735. doi: 10.1371/journal.ppat.1010735 (PMC9810172; doi:10.1371/journal.ppat.1010735)
Supplement: S1 Table — Individual results at the end-of-phase-1(P1) are given for animals with high toxemia, low ratios, and early time-to-death (fast progression) and animals with low toxemia, high ratios, and later time-to-death. Median, upper quartile, and lower quartile, determined in JMP. P-values comparing fast and slow progression obtained by Mann-Whitney-Wilcoxon rank sum (non-parametric) test. Levels for survivor shown separately. *Still in phase-1, excluded. **Less than the limit of detection (<LOD) for PA83 (1.22 ng/mL) given a value at ½ times the LOD for PA83 (0.61 ng/mL) for calculations. (PDF) [file ppat.1010735.s009.pdf]

S1 Table. End-of-phase-1 toxins, bacteremia, and toxin ratios. Individual results at the end-of-phase-1(P1) are given for animals with high toxemia, low ratios, and early time-to-death (fast progression) and animals with low toxemia, high ratios, and later time-to-death. Median, upper quartile, and lower quartile, determined in JMP. P-values comparing fast and slow progression obtained by Mann-Whitney-Wilcoxon rank sum (non-parametric) test. Levels for survivor shown separately. *Still in phase-1, excluded. **Less than the limit of detection (<LOD) for PA83 (1.22 ng/mL) given a value at ½ times the LOD for PA83 (0.61 ng/mL) for calculations.

Animal ID	Time to Death (hours)	End-of-P1 Time Point	Total-PA (ng/mL)	PA83 (ng/mL)	Total LF (ng/mL)	Lethal Toxin (ng/mL)	Total EF (ng/mL)	Edema Toxin (ng/mL)	Bact (cfu/mL)	PA/LF Ratio	LF/LTx Ratio	LF/EF Ratio	EF/ETx Ratio	PA/ETx Ratio	Phase
1) C58282*	48.5	48 h NA (36h shown)	9.11	<LOD	2.99	0.865	0.026	0.0045	5.3E+02	3.0	3.5	115.0	5.8	2024	P1*
2) C59621	48.9	42 h	333	67.4	57.8	23.7	20.7	1.872	2.9E+06	5.8	2.4	2.8	11.1	178	EOP1-P2
3) C60763	54.7	30 h	326	45.2	50.9	15.3	2.6	0.576	9.4E+05	6.4	3.3	19.6	4.5	566	EOP1-P2
4) C58176	55.2	30 h	188	25.6	200	81.7	2.68	0.635	8.3E+06	0.9	2.4	74.6	4.2	296	EOP1-P2
5) C58003	57.3	42 h	1143	163	300	169	23.7	5.64	5.9E+06	3.8	1.8	12.7	4.2	203	EOP1-P2
6) C59656	57.9	48 h	731	182	75.2	27.7	28	4.97	3.6E+06	9.7	2.7	2.7	5.6	147	EOP1-P2
7) C58214	58.4	48 h	509	97.6	56.2	21	5.58	0.307	1.3E+06	9.1	2.7	10.1	18.2	1658	EOP1-P2
8) C60732	59	36 h	1189	176	313	165	39.2	7.1	7.4E+06	3.8	1.9	8.0	5.5	167	EOP1-P2
9) C59669	64.8	48 h	3035	621	1038	573	208	38.9	3.2E+07	2.9	1.8	5.0	5.3	78	EOP1-P2
10) C57161	66.9	48 h	2221	516	366	245	53.4	15	7.2E+06	6.1	1.5	6.9	3.6	148	EOP1-P2
11) C58170	74.1	48 h	102	12.1	38.4	5.53	2.16	0.134	3.3E+05	2.7	6.9	17.8	16.1	761	EOP1-P2
	Median		620	130	138	54.7	22.2	3.4	4.8E+06	4.8	2.4	9.0	5.4	190	
	lower quartile		292	40.3	54.9	19.6	2.7	0.51	1.2E+06	2.9	1.8	4.4	4.2	148	
	upper quartile		1447	266	326	188	42.8	9.1	7.6E+06	7.1	2.9	18.2	12.3	615	
12) C59619	76.4	36 h	368	36.2	32.5	12.5	0.661	0.278	1.8E+05	11.3	2.6	49.2	2.4	1324	EOP1-P2
13) C58135	90.4	60 h	328	33.1	41.5	13	0.922	0.153	6.2E+04	7.9	3.2	45.0	6.0	2144	EOP1-P2
14) C57793	91.1	48 h	133	8.8	23.8	4.55	0.401	0.038	6.1E+04	5.6	5.2	59.4	10.6	3500	EOP1-P2
15) C58167	91.7	36 h	89.7	7.6	26.3	7.94	0.743	0.242	1.5E+05	3.4	3.3	35.4	3.1	371	EOP1-P2
16) C57998	100.8	48 h	41	<LOD**	16.2	6.32	0.064	0.001	5.6E+03	2.5	2.6	253.1	64.0	41000	EOP1-P2
17) C59617	105.1	48 h	54.2	<LOD**	12.4	1.91	0.032	0.002	3.3E+03	4.4	6.5	387.5	16.0	27100	EOP1-P2
18) C58159	109	48 h	121	7.89	23.8	8.76	0.231	0.0057	3.1E+04	5.1	2.7	103.0	40.5	21228	EOP1-P2
19) C58122	115.1	48 h	152	<LOD**	22	9.03	0.097	0.0048	3.6E+03	6.9	2.4	226.8	20.2	31667	EOP1-P2
20) C57997	125.9	48 h	165	11.8	15.6	8.62	0.166	0.020	1.1E+04	10.6	1.8	94.0	8.3	8250	EOP1-P2
21) C58156	140.1	48 h	200	<LOD**	26.1	10.3	0.081	0.0043	7.8E+03	7.7	2.5	322.2	18.8	46512	EOP1-P2
22) C59618	201.9	42 h	29.6	<LOD**	12.4	1.53	0.046	0.0015	9.4E+03	2.4	8.1	269.6	30.7	19733	EOP1-P2
	Median		133	7.6	23.8	8.6	0.17	0.0057	1.1E+04	5.6	2.7	103	16	19733	
	lower quartile		54.2	0.61	15.6	4.6	0.064	0.0020	5.6E+03	3.4	2.5	49.2	6.0	2144	
	upper quartile		200	11.8	26.3	10.3	0.66	0.15	6.2E+04	7.9	5.2	270	30.7	31667	
	Fast-v-Slow p-value		0.0035	0.0003	0.0002	0.0011	0.0001	0.0003	0.0001	0.5495	0.149	0.0004	0.098	0.0004	
23) C58174	Survivor	42 h	33.9	<LOD	11.1	3.25	0.040	0.0023	6.4E+03	3.05	3.42	278	17.4	14739	EOP1-P2
